# Reproducibility of preclinical animal research improves with heterogeneity of study samples

**DOI:** 10.1371/journal.pbio.2003693

**Published:** 2018-02-22

**Authors:** Bernhard Voelkl, Lucile Vogt, Emily S. Sena, Hanno Würbel

**Affiliations:** 1 Division of Animal Welfare, VPH Institute, Vetsuisse Faculty, University of Bern, Bern, Switzerland; 2 Centre for Clinical Brain Sciences, Chancellors Building, University of Edinburgh, Edinburgh, United Kingdom; University of Amsterdam, Netherlands

## Abstract

Single-laboratory studies conducted under highly standardized conditions are the gold standard in preclinical animal research. Using simulations based on 440 preclinical studies across 13 different interventions in animal models of stroke, myocardial infarction, and breast cancer, we compared the accuracy of effect size estimates between single-laboratory and multi-laboratory study designs. Single-laboratory studies generally failed to predict effect size accurately, and larger sample sizes rendered effect size estimates even less accurate. By contrast, multi-laboratory designs including as few as 2 to 4 laboratories increased coverage probability by up to 42 percentage points without a need for larger sample sizes. These findings demonstrate that within-study standardization is a major cause of poor reproducibility. More representative study samples are required to improve the external validity and reproducibility of preclinical animal research and to prevent wasting animals and resources for inconclusive research.

## Author summary

Preclinical animal research is mostly based on studies conducted in a single laboratory and under highly standardized conditions. This entails the risk that the study results may only be valid under the specific conditions of the test laboratory, which may explain the poor reproducibility of preclinical animal research. To test this hypothesis, we used simulations based on 440 preclinical studies across 13 different interventions in animal models of stroke, myocardial infarction, and breast cancer and compared the reproducibility of results between single-laboratory and multi-laboratory studies. To simulate multi-laboratory studies, we combined data from multiple studies, as if several collaborating laboratories had conducted them in parallel. We found that single-laboratory studies produced large variation between study results. By contrast, multi-laboratory studies including as few as 2 to 4 laboratories produced much more consistent results, thereby increasing reproducibility without a need for larger sample sizes. Our findings demonstrate that excessive standardization is a source of poor reproducibility because it ignores biologically meaningful variation. We conclude that multi-laboratory studies—and potentially other ways of creating more heterogeneous study samples—provide an effective means of improving the reproducibility of study results, which is crucial to prevent wasting animals and resources for inconclusive research.

## Introduction

Reproducibility of results from preclinical animal research is alarmingly low, and various threats to reproducibility have been proposed, including a lack of scientific rigor, low statistical power, analytical flexibility, and publication bias [1–8]. All of these biases undermine the scientific validity of findings published in the scientific literature; however, empirical evidence demonstrating a causal link between any of these aspects and poor reproducibility in preclinical research is critically lacking. Moreover, an important aspect that has been almost completely overlooked so far is the rigorous standardization of animal experiments. Importantly, while all other sources of poor reproducibility mentioned above represent violations of good laboratory practice, standardization is considered good laboratory practice. Therefore, both genetic standardization (animals) and environmental standardization (housing and husbandry) are explicitly recommended by laboratory animal science textbooks [9] and are taught in laboratory animal science courses as a means to guarantee both precision and reproducibility. However, standardization renders study populations more homogenous and the results more specific to the specific standardized study conditions. Therefore, contrary to the common belief that standardization guarantees reproducibility (e.g., [9]), both theoretical [10–12] and empirical [13–17] evidence indicate that rigorous standardization may generate spurious results that are idiosyncratic to the specific standardized conditions under which they were obtained, thereby causing poor reproducibility. This is because the response of an animal to an experimental treatment (e.g., a drug) often depends on the phenotypic state of the animal, which is a product of the genotype and the environmental conditions. Therefore, phenotypic plasticity caused by gene-by-environment (G × E) interactions determines the range of variation (reaction norm) of an animal’s response [18]. Instead of incorporating such natural biological variation in the experimental design, laboratory animal scientists consider this variation as a nuisance, which they aim to eliminate through rigorous standardization of both genotype and environmental conditions [9]. However, because laboratories differ in many environmental factors that affect the animals’ phenotype (e.g., noise, odors, microbiota, or personnel [13,19]), animals will always differ between laboratories due to G × E interactions, and the variation of phenotypes between laboratories is generally much larger than the variation within laboratories. This implies that whenever a study is replicated in a different laboratory, a distinct sample of phenotypes will be tested. Therefore, instead of indicating that a study was biased or underpowered, a failure to reproduce its results might rather indicate that the replication study was testing animals of a different phenotype [12,16]. Nevertheless, rigorously standardized single-laboratory studies continue to be the gold standard approach to animal research from basic exploratory research to late-phase preclinical testing.

A landmark study that brought this problem to the attention of the scientific community for the first time was a multi-laboratory study by Crabbe and colleagues [13] investigating the confounding effects of the laboratory environment and G × E interactions on behavioral strain differences in mice. Despite rigorous standardization of housing conditions and study protocols across 3 laboratories, systematic differences were found between laboratories, as well as significant interactions between genotype and laboratory. The most direct way to account for such between-laboratory variation is the use of multi-laboratory study designs. Such study designs are common in medical research, especially for Phase III clinical trials [20], and increasingly also in psychological research [21,22]. While clinical multicenter studies are often motivated by the need to recruit large samples, their potential for detecting confounding effects has been recognized by the research community [23–25]. However, in preclinical animal research, the confounding effect of the laboratory is likely to be much stronger because laboratory standards of housing and care strongly affect the animals’ phenotype. Nevertheless, multi-laboratory studies are still very uncommon in preclinical animal research, despite recent initiatives [26,27] promoting their implementation. The aim of this study is, therefore, to assess how the heterogenization of study samples through multi-laboratory study designs affects the outcome of preclinical animal studies, with the hypothesis that it improves the accuracy and reproducibility of the results.

## Results

To investigate how multi-laboratory designs alter the outcome and reproducibility of preclinical animal studies, we simulated single-laboratory and multi-laboratory studies based on published data of preclinical research obtained through the Collaborative Approach to Meta-Analysis and Review of Animal Data from Experimental Studies (CAMARADES) database [28,29]. In a first step, we selected 50 independent studies on the effect of therapeutic hypothermia on infarct volume in rodent models of stroke. In a second step, we replicated the same analysis with 12 further interventions in animal models of stroke, myocardial infarction, and breast cancer. For the sake of clarity, and to reflect the progression of this study, we will first present the analysis of the hypothermia data in full detail, followed by a summary of the analysis of the 12 replicate data sets.

**Fig 1:**
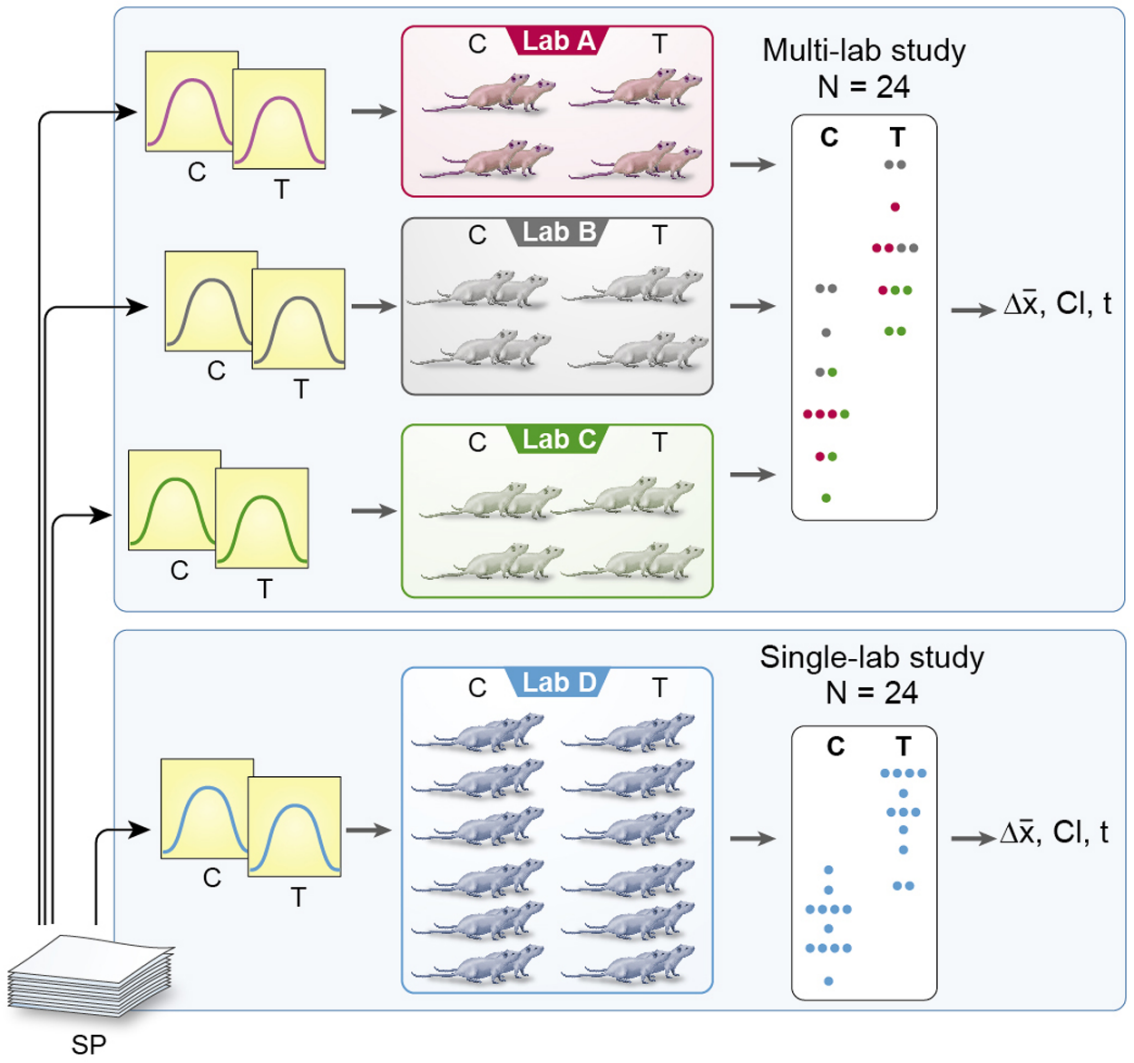
Sampling scheme for simulated single-lab and multi-lab studies. For a single-lab study, 1 original study is randomly selected from the study pool, and response values for control and treatment groups are generated by sampling from a Normal distribution with parameters as reported in the original study. For the multi-lab study, several original studies are selected, and values are sampled proportionate from the corresponding distributions. C, control group; SP, study pool; T, treatment group.

A random-effect meta-analysis of the 50 studies on hypothermia yielded an estimated mean reduction of infarct volume by hypothermia of 47.8% (95% confidence interval [CI_95_] = 40.6%–55.0%). For the simulation of single-laboratory versus multi-laboratory studies, we took this estimate as our estimate of the “true” effect. The existence of such an effect is corroborated by the efficacy of hypothermia in clinical settings [30,31]. This conjecture allowed us to compare the performance of different study designs by assessing how often and how accurately the simulated studies predicted that effect. Specifically, we compared effect size estimates and inferential statistics of single-laboratory studies to multi-laboratory studies including 2, 3, or 4 randomly selected laboratories, using the same sample size for all designs (Fig 1).

Given typical sample sizes in early preclinical animal research, we first simulated studies with a sample size of 12 animals per treatment group (*N* = 24). By randomly selecting 1 study and sampling 12 values from a Normal distribution with parameters as reported for the control group, and likewise sampling another 12 values with parameters as reported for the treatment group, we calculated an effect size estimate (mean difference) and a corresponding CI_95_ (Fig 1). Repeating this procedure 10^5^ times, we found that, of such simulated single-laboratory studies, the CI_95_ captured the true effect size (i.e., the summary effect size of the meta-analysis) in only 47.9% of the cases (coverage probability [pc] = 0.48), and inferential tests failed to find a significant effect in 17.6% of the cases (false negative rate [FNR] = 0.18). Therefore, although the studies were sufficiently powered (>0.8) to detect a treatment effect, single-laboratory studies failed to predict the true effect size accurately in more than half of the cases.

**Fig 2:**
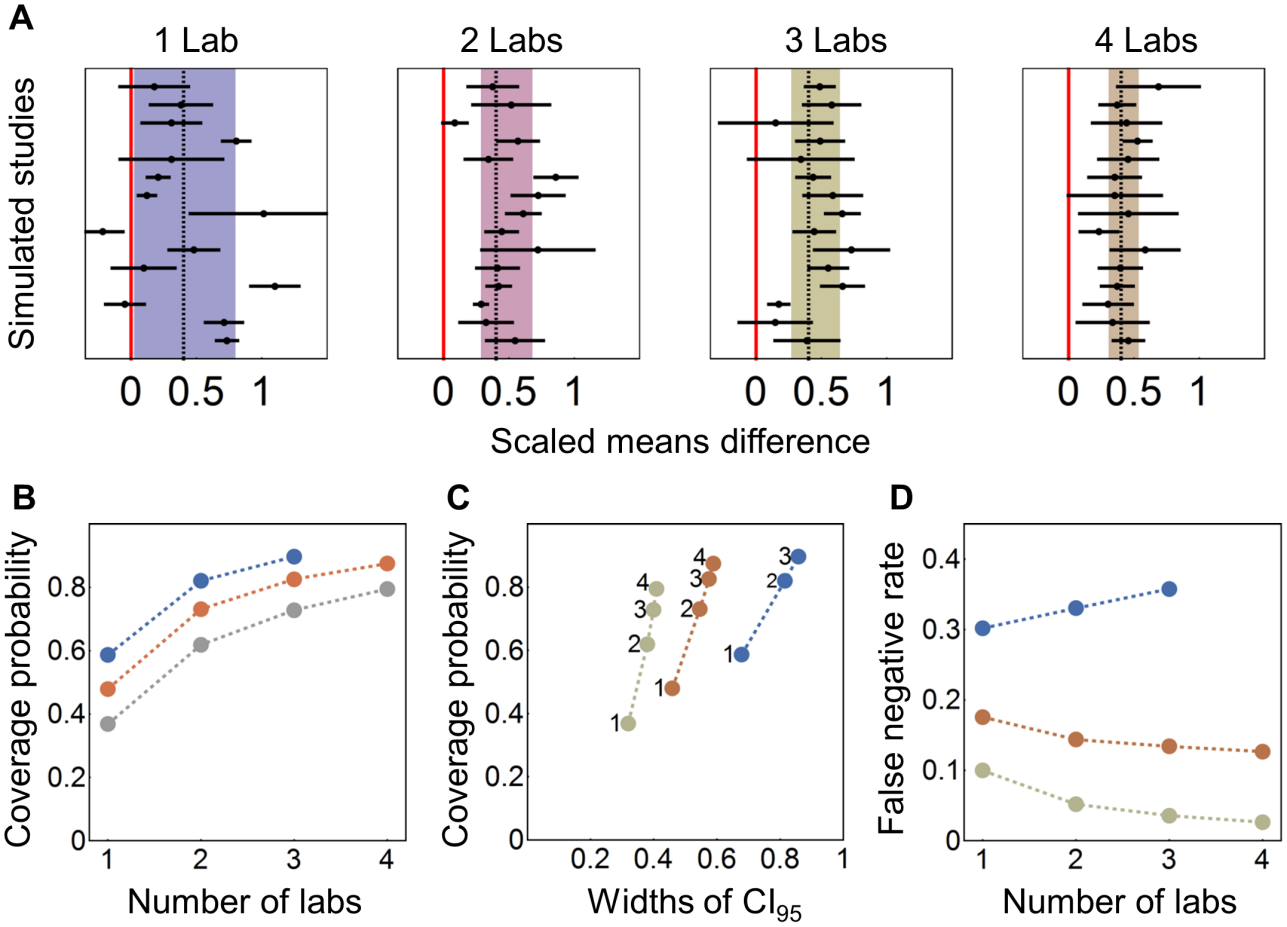
Results of resampling from studies on hypothermia in rodent models of stroke. (A) Forest plot of 15 randomly selected simulated studies for the 1-, 2-, 3-, and 4-lab scenario and *N* = 24; dashed line: estimated true effect; shaded area: 95% CI for the effect size estimate based on the sampled studies. The red line indicates a null effect (effect size of 0). (B) pc plotted against the number of participating laboratories for *N* = 12 (blue), *N* = 24 (orange), and *N* = 48 (grey). (C) pc plotted against the average width of the 95% CI. (D) False negative rate plotted against number of laboratories. pc, coverage probability.

To simulate multi-laboratory designs, 2, 3, or 4 different studies were randomly drawn from the pool of 50 studies, and proportionate numbers of sample values for both control and treatment group were generated to run these multi-laboratory studies with the same overall sample size as the single-laboratory studies (Fig 1). For the 2-laboratory design, pc increased to 0.73, for the 3-lab design to 0.83, and for the 4-laboratory design to 0.87, while the FNR decreased to 0.14, 0.13, and 0.13, respectively. The increase in pc with increasing numbers of laboratories is a result of increased accuracy and reduced variation between effect size estimates. These findings are illustrated in Fig 2A, showing exemplary forest plots based on 15 randomly selected simulations for each study design. As illustrated by the first panel, effect size estimates of single-laboratory studies varied substantially, ranging from detrimental effects of hypothermia on infarct volume (effect size <0) to the complete abolition of infarct through hypothermia (effect size ≈ 1). By contrast, multi-laboratory studies including 4 laboratories produced effect size estimates very close to the true effect. The decrease of between-study variation in effect size estimates with increasing number of laboratories per study is illustrated by the width of the summary confidence interval (shaded area), which reflects the reproducibility of the results of the sampled studies.

We repeated this analysis with a smaller (*N* = 12) and a larger (*N* = 48) overall sample size to cover a range of sample sizes commonly encountered in in-vivo research. This range would comprise 7,339 (84%) of the 8,746 preclinical studies in the CAMARADES database. For *N* = 12, we only investigated the 1-, 2-, and 3-laboratory conditions but not the 4-laboratory condition because 12 animals cannot be distributed evenly over 4 laboratories and 2 experimental conditions. For all 3 sample sizes, we found an increase in pc with increasing number of participating laboratories (Fig 2B). Plotting pc against the mean width of the CI_95_ (Fig 2C) shows that the increase in pc was associated with an increase in the width of the CI_95_ estimates, yet the trade-off was reduced with increasing sample size (indicated by the steeper slopes for larger sample sizes in Fig 2C). In line with this, increasing the number of participating laboratories affected the FNR, depending on sample size. Whereas for larger sample sizes (*N* = 24 and *N* = 48) the FNR decreased with increasing number of laboratories, this trend was reversed for *N* = 12, with the FNR increasing from 0.30 for 1 laboratory to 0.36 for 3 participating laboratories (Fig 2D). Fig 2D suggests that a divide exists somewhere near FNR of 0.2: when sample sizes were large enough for the single-laboratory design to achieve an FNR of 0.2 (reflecting statistical power of 0.8), multi-laboratory designs reduced the FNR further. In contrast, when statistical power of the single-laboratory design was below 0.8, multi-laboratory designs can lead to an increase of the FNR.

**Fig 3:**
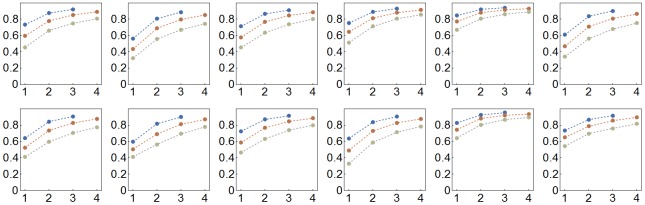
Coverage probability plotted against the number of participating laboratories for *N* = 12 (blue), *N* = 24 (orange), and *N* = 48 (grey) for simulated studies for 12 additional intervention studies of mouse models of stroke, myocardial infarction, and breast cancer. First row: tPA, trastuzumab, FK506, rosiglitazone 2, IL-1RA, cardiosphere DC; second row: estradiol, human MSC, MK-80, TMZ, c-kit CSC, rat BMSC (see Table A in S1 Text for details). BMSC, bone marrow stem cell; CSC, cardiac stem cell; DC, derived cell; IL1-RA, interleukin 1 receptor antagonist; MSC, mesenchymal stem cell; TMZ, temozolomide; tPA, tissue plasminogen activator.

To determine whether these findings generalize across experimental treatments, we replicated this simulation study based on data for a further 12 interventions in animal models of stroke, myocardial infarction, and breast cancer (*N* = 20–58 studies per intervention; Table A in S1 Text). In all cases, we found an increase in pc with increasing number of participating laboratories (Fig 3). We also replicated the finding that the FNR generally decreases in multi-laboratory designs when statistical power is high but may increase when statistical power is low, though the exact level of statistical power above which FNR decreases in multi-laboratory designs may vary (Fig D in S1 Text).

**Fig 4:**
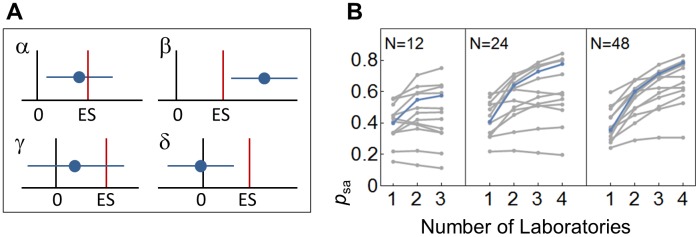
Proportion of studies reporting significant and accurate effects. (A) Schematic of study outcomes. A study reporting both ES estimates and inferential significant statements can lead to 1 of 4 outcomes. (α) The reported CI for the ES estimate (horizontal blue line) includes the true ES, and the CI is not including 0, suggesting the existence of an effect; (β) the CI covers neither 0 nor the true ES, suggesting the existence of an effect, though its magnitude is either over- or underestimated; (γ) the CI covers the true effect but also 0—in this case “no significant” effect would be reported, and the ES estimate would be ignored or treated as nonrelevant (which is often the case in underpowered studies); (δ) the CI includes 0 but not the true ES, leading again to a “nonsignificant” result. Based on this, we can calculate the ratio of studies accurately estimating the true ES as p_sa_ = α / (α + β + γ + δ). (B) p_sa_ based on 10^5^ simulated samples for the hypothermia treatment of stroke (blue) and 12 further interventions (grey) for total sample sizes of *N* = 12, 24, and 48 subjects and *k* = 1–4 laboratories. ES, effect size.

Because it is common practice to interpret effect sizes conditional on statistical significance (for a critique of this, see, e.g., [3,32]), we calculated the proportion of studies reporting a “statistically significant” and “accurate” effect size estimate with a CI covering the true effect but not 0, p_sa_ (see Fig 4A for definition), which can be regarded as a measure of external validity in an ideal world without publication bias. As shown in Fig 4B, the external validity in terms of the proportion of statistically significant and accurate effect size estimates (p_sa_) increased substantially in almost all cases (Fig 4B). Increasing the number of participating laboratories introduced heterogeneity and increased the total variance. In the absence of such effects, multi-laboratory designs would not substantially alter effect size estimates and statistical inference. However, heterogeneity among laboratories was large in all 13 data sets (median *I*^2^ = 85%, range: 42%–97%, where *I*^2^ is the ratio of excess dispersion to total dispersion; Table 1, Table B in S1 Text). In fact, taking a reaction norm perspective on animal traits, such environment-dependent differences and the resulting interactions are expected to be ubiquitous [12,16].

**Table 1:** Definitions of key terms used in this manuscript.

Key term	Definition
Reproducibility	The similarity of outcomes between replicate studies. This can be measured, e.g., by the CI_95_ of the mean effect size estimates of a sample of replicate studies (depicted by the shaded area in Fig 2A).
FNR	False negative rate: proportion of true positives that yield negative test outcomes. FNR = false negative ÷ (true positive + false negative).
FPR	False positive rate: proportion of true negatives that yield positive test outcomes. FPR = false positive ÷ (false positive + true negative).
DOR	Diagnostic odds ratio: ratio of the odds of the test being positive in the case of a true positive relative to the odds of the test being positive in the case of a true negative. DOR = (true positive ÷ false positive) ÷ (false negative ÷ true negative).
pc	Coverage probability: the probability with which the CI_95_ of an effect size estimate includes the true effect size.
p_sa_	The proportion of studies reporting both a significant effect for α = 0.05 and a CI_95_ for the effect size estimate that includes the true effect.
*I*^2^	*I*^2^ is a descriptive statistic of the ratio of excess dispersion to total dispersion. *I*^2^ = (Q − df ÷ Q) × 100%, where Q is the weighted sum of squares of study effect sizes and df gives the degrees of freedom.

Abbreviations: CI_95_, 95% confidence interval; DOR, diagnostic odds ratio; FNR, false negative rate; FPR, false positive rate; pc, coverage probability.

## Discussion

Using simulated sampling, we compared the outcomes of single- and multi-laboratory studies, using the same overall number of animals, in terms of their accuracy of effect size estimates (pc) and FNR. For these simulations, we chose to use a large sample of published data from preclinical studies to guarantee that the results reflect real-life conditions. We found that pc increased substantially with the number of participating laboratories, without causing a need for larger sample sizes. This demonstrates that using more representative study samples through multi-laboratory designs improves the external validity and reproducibility of preclinical animal research.

Although higher pc and greater external validity come at the cost of higher uncertainty (i.e., wider CIs), this simply reflects the true uncertainty that exists when certain sources of variation are either unknown or unavoidable, which is usually the case in animal research. Of course, we cannot exclude some bias among the study samples used for our simulation approach (due to, e.g., lack of scientific rigor, publication bias). Both lack of scientific rigor [33] and publication bias [34] have been found to inflate summary effect sizes in meta-analyses. However, although this remains to be examined further, there is currently no evidence to suggest that accounting for such risks of bias would reduce the variation among replicate studies, thereby invalidating our findings. Rather, our results suggest that eliminating these and other risks of bias (e.g., low statistical power, analytical flexibility) is not sufficient to guarantee reproducibility; the results will remain idiosyncratic to the specific laboratory conditions unless these conditions are varied. Importantly, we see that increasing sample size to increase statistical power does not help but makes things even worse: it produces results that are more precise (smaller CIs) but less accurate (decreased pc) and therefore less reproducible. Relying on more representative study samples to improve the accuracy of effect size estimates may therefore be a critical step on the way out of the current reproducibility crisis.

Taken together, our results indicate that multi-laboratory designs—and possibly other means of systematic heterogenization of study samples—will increase the accuracy of results and decrease inference errors, as long as the studies are sufficiently powered. As a consequence of this, results will gain external validity and therefore be more likely to be reproducible. Importantly, these improvements require neither many participating laboratories nor larger sample sizes. In fact, the greatest improvement in pc was observed between single-laboratory studies and studies involving 2 laboratories. As a rule of thumb, we suggest that multi-laboratory designs can improve inference and accuracy of effect size estimates, whenever sample size is large enough to achieve statistical power of at least 0.8 for a 1-way ANOVA design (i.e., a single-lab study). This suggestion is based on the finding that the trade-off between increased pc and increased uncertainty (the width of the CIs) with increasing numbers of laboratories may result in an increased FNR, which may override the positive effect of increased pc when sample size is too small.

The effects that we show here are consistent with findings reported by IntHout and colleagues [35], who compared inference errors of a single highly powered study to those of several low-powered studies, combined in a random-effects meta-analysis. These authors showed that even low levels of heterogeneity can lead to increased false positive rates (FPRs) of single-laboratory studies, while meta-analyses based on even just 2 randomly selected studies lead to notably reduced FPRs. Comparing the effect of meta-analyses comprising 2 or 3 studies, InHout and colleagues found that the largest reduction in the FPR was observed when moving from the interpretation of 1 to 2 studies, while meta-analyses with 3 studies performed very similarly to those with only 2 studies [35]. This, too, is in line with our findings that the largest increase in pc is found when contrasting single-laboratory studies with a multi-laboratory study involving 2 participating laboratories. Furthermore, it mirrors recommendations issued by the Food and Drug Administration (FDA) [36] and the European Agency for the Evaluation of Medicinal Products (EMEA) [37] to replicate studies at least once (*N* = 2).

Besides known differences between the studies included in our analysis, such as the species or strain of animals (i.e., genotype) or reported differences in animal husbandry and experimental procedures, sources of variation included also many unknown and unknowable differences, such as the influence of the experimenter [38,39] or the microbiome [40], as well as subtle differences in visual, olfactory, and auditory stimulation. All those factors might affect treatment effects. Multi-laboratory designs are ideal to account for all of these sources of between-laboratory variation and should therefore replace standardized single-laboratory studies as the gold standard for late-phase preclinical trials [27].

However, logistic limitations may render multi-laboratory studies unsuitable for earlier, more basic types of research. One approach that was recently proposed is to statistically account for between-laboratory variation in single-laboratory studies by including a Treatment by Laboratory (T × L) interaction term as a random factor in the analysis [16]. This “Random Lab Model” (RLM) approach generates an adjusted yardstick against which treatment effects are tested in single-laboratory studies. A recent analysis of multi-laboratory data sets indicated that T × L adjustment can reduce spurious results and improve reproducibility considerably without losing much statistical power [16]. Compared with simply lowering the *p*-value of statistical significance across the board to, e.g., 0.005 as proposed by others [41,42], T × L adjustment is more specific because it takes the true heterogeneity among different laboratories into account. However, the RLM approach depends on reliable estimates of T × L interaction, which for most animal studies are not readily available. Whether the strength of this interaction can at least be roughly estimated for specific research fields as proposed by Kafkafi and colleagues [16] remains to be tested empirically.

Because multi-laboratory studies are logistically demanding and may not be appropriate for more basic or exploratory studies, and because statistical approaches may be worrisome because of questionable assumptions, an alternative approach would be to systematically heterogenize experimental conditions, thereby mimicking multi-laboratory studies within single-laboratory studies [15]. For example, Karp and colleagues [17] found considerable phenotypic variation between different batches of knockout mice tested successively in the same laboratory. Therefore, batch heterogenization might be a useful starting point for within-lab heterogenization. A proof-of-concept study demonstrated that heterogenization based on age and housing condition of mice can improve the reproducibility of results [14], but an experimental test indicated that such simple forms of heterogenization may not be effective enough to account for the large variation between replicate studies in different laboratories [43]. In the present study, the heterogeneity among the studies used for the simulations comprised both environmental differences between laboratories and genetic differences between the different strains or—in some cases—different species used. The heterogeneity found here may, therefore, be larger than in a planned multi-laboratory study based on a specific strain of animals and harmonized environmental conditions. However, as shown here, such variation is real in preclinical research, and the evidence base generated by meta-analysis commonly includes such variation. An important future goal will therefore be to find practicable ways to mimic between-laboratory variation within single-laboratory studies using controlled, systematic variation of relevant genetic and environmental variables.

Standardization is often promoted also for ethical reasons because standardization reduces variation in experimental results, and therefore fewer animals are needed per experiment to achieve a desired level of statistical power. Using as few animals as possible for animal research is an important goal of the 3Rs principles [44]. However, our findings show that reducing animals per experiment through standardization may be short sighted because it means trading animals against the external validity and reproducibility of experimental results. Poor external validity and poor reproducibility question the benefit of the research in the harm-benefit analysis of animal experiments, which could mean that although fewer animals may be used in a standardized experiment, they may be wasted for inconclusive research [45]. As a consequence, more replicate experiments may be needed—and therefore overall more animals—to answer a given research question conclusively, which is clearly at odds with the 3Rs principles.

## Materials and methods

### Data acquisition and simulated sampling

Parameter estimates for the simulations were extracted from the CAMARADES [27,46] database, based on a list of a priori inclusion and exclusion criteria (Fig A in S1 Text, Table A in S1 Text). All included studies were of a 1-way ANOVA design, reporting mean estimates for a control group and a treatment group along with standard deviations, but they differed in several aspects of study protocol, including species or strain of animals, experimental procedure, and outcome assessment. We therefore scaled the reported parameter values for each study by dividing them by the mean estimate for the control group of that study. In order to simulate a single-laboratory study, we randomly selected 1 study from the study pool and sampled 6, 12, or 24 values from a Normal distribution with according parameter values for the control group and another 6, 12, or 24 values from a Normal distribution with according parameter values for the treatment group. For multi-laboratory studies with *k* laboratories, we randomly selected *k* studies from the study pool and sampled 1/*k* of values from the distributions of each respective study. For each simulated study, we calculated the mean difference as effect size estimate and performed a 1-way ANOVA for the 1-laboratory case and a fixed-effect 2-way ANOVA with treatment and laboratory as main effects and α = 0.05 for inference for the multi-laboratory setting. An extended discussion of alternative approaches for analyzing data of multi-lab studies (pooled *t* tests, mixed-effect linear models) is given below. The ANOVA outcome allowed us to estimate the FNR—i.e., the proportion of cases in which the F-ratio test of the ANOVA did not indicate a significant difference between groups. To assess the FPR (i.e., the proportion of cases in which the F-ratio test of the ANOVA did indicate a significant difference between groups, even though there was none), we ran a second set of simulations in which again we randomly selected original studies, but for which parameter values for both control and treatment group were drawn from the same Normal distribution with mean and standard deviation being set to the mean of the reported values for treatment and control group. The FPR of the 2-way ANOVA stayed relatively close to 0.05 under all conditions (Fig B in S1 Text, Fig C in S1 Text), corroborating the suitability of the test. As a consequence, changes in the diagnostic odds ratio (DOR; Fig B in S1 Text, Fig C in S1 Text) were mainly driven by the FNR. Simulations were first run in R 3.2.2. by LV and independently replicated by BV using Mathematica 10.1. (Wolfram Research, www.wolfram.com; see S1 and S2 Text for pseudocode and program code). Reported numbers and figures are based on the simulations run in Mathematica. Random-effect meta-analyses on the original data sets were carried out using the R package metafor1.9–9 [47] with restricted maximum likelihood estimators.

### Analysis of multi-laboratory studies

The design of multi-lab studies presented in this analysis is a 2-way ANOVA design with one factor being the treatment with 2 levels—treatment or control—and the other factor being the laboratory at which subjects were housed and tested. The interaction term was not included. In the case of a single-lab study, this simplifies to a 1-way ANOVA design. Different analysis schemes have been used in the past, and battles about the appropriate analysis have been fought elsewhere [25,48,49]. The statistical analysis of multi-laboratory studies is not the topic of this manuscript, and we deliberately abstained from discussing this issue in the main text (but see S1 Text for an extended discussion). For didactical clarity, we have chosen a fixed-effect ANOVA, though with respect to our focus, the same outcomes would be retrieved if we simply performed a *t* test on the pooled (and scaled) data—as it was sometimes done in the past [49,50, but see 32,51 for a critique]—or if we treated laboratory as a random factor in a linear mixed-effect model as it is more recently advocated [23,51–53] (Fig D in S1 Text).

## Supporting information

S1 Text Supporting information, including inclusion/exclusion criteria for data sets, data set summaries, supporting discussion of the inference method, supporting data, and annotated code. (PDF)

S2 Text Mathematica code for simulated sampling. (PDF)

S1 Code Mathematica notebook with code for simulated sampling. (NB)

S1 Data Data extracted from the CAMARADES database and used in this study. CAMARADES, Collaborative Approach to Meta-Analysis and Review of Animal Data from Experimental Studies.
(CSV)

## Abbreviations

BMSC: bone marrow stem cell
CAMARADES: Collaborative Approach to Meta-Analysis and Review of Animal Data from Experimental Studies
CI_95_: 95% confidence interval
CSC: cardiac stem cell
DC: derived cell
DOR: diagnostic odds ratio
EMEA: European Agency for the Evaluation of Medicinal Products
FDA: Food and Drug Administration
FNR: false negative rate
FPR: false positive rate
G × E: gene by environment
IL1-RA: interleukin 1 receptor antagonist
MSC: mesenchymal stem cell
pc: coverage probability
RLM: Random Lab Model
T × L: treatment by laboratory
TMZ: temozolomide
tPA: tissue plasminogen activator
