# Supplementary material for: Reproducibility of preclinical animal research improves with heterogeneity of study samples
Source: PLoS Biol. 2018 Feb 22;16(2):e2003693. doi: 10.1371/journal.pbio.2003693 (PMC5823461; doi:10.1371/journal.pbio.2003693)
Supplement: S1 Text — (PDF) [file pbio.2003693.s001.pdf]

Reproducibility of pre-clinical animal research improves with heterogeneity of study samples

Bernhard Voelkl*, Lucile Vogt*, Emily S. Sena, Hanno Würbel

Supporting Text

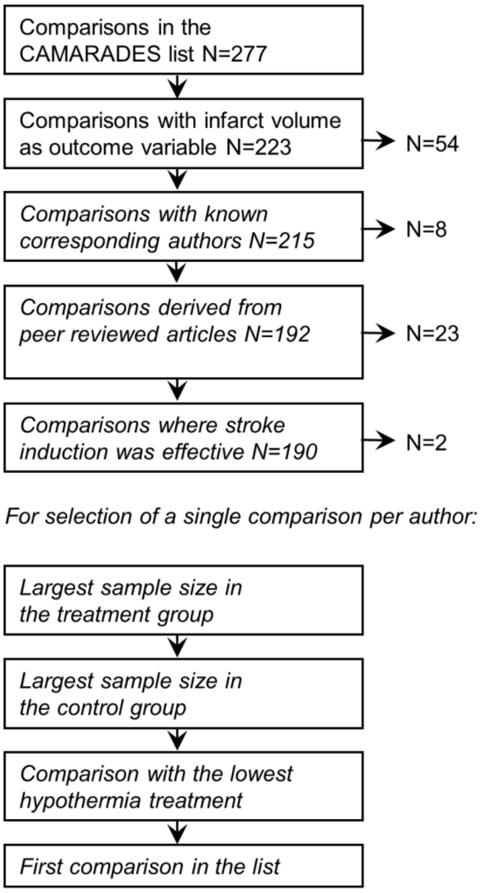

Figure A: Inclusion/exclusion criteria for the data set of hypothermia studies. The CAMARADES database contains a total of 277 comparisons between a control and a hypothermia treated group. We included only comparisons where the outcome variable was infarct volume (N=223) with a known author for correspondence (N=215), derived from a peer-reviewed article (N=192) and for which an infarct was present (i.e. induction of the lesion was successful. N=190). In order to ensure that comparisons were independent (i.e. they came from different laboratories), we classified comparisons by corresponding author and applied an additional set of inclusion criteria to obtain only one comparison per corresponding author. The 190 comparisons were published by 50 different corresponding authors. When more than one comparison was published by the same author, we selected the comparison with the largest sample size in the treatment group. If more than one study had shared maximum sample size, the study with the largest sample size in the control group was selected. When more than one study had the same sample sizes for both groups, we selected the comparison for which the lowest hypothermia temperature was used. Finally, if there were still more than one comparison by corresponding author, we selected the first comparison on the list.

	Intervention	Outcome	Species	restrict	N	Power: Median	Quartiles
0	hypothermia	Infarct volume	Rat	Yes	50	0.95	0.82-0.99
1	tPA	Infarct volume	Rat	Yes	57	0.14	0.12-0.17
2	Trastuzumab	Tumour volume ratio	Mouse	No	58	0.77	0.55-0.87
3	FK506	Infarct volume	Rat	Yes	31	0.95	0.92-0.96
4	Rosiglitazone 2	Infarct volume	Rodent	No	21	0.93	0.83-0.96
5	IL-1RA	Infarct volume	Rodent	No	37	0.42	0.34-0.57
6	Cardiosphere DC	EF (%)	Rodent	Yes	35	0.98	0.97-0.99
7	Estradiol	Infarct volume	Rat	Yes	24	0.57	0.37-0.63
8	Human MSC	Infarct volume	Rat	No	26	0.56	0.56-0.78
9	MK-801	Infarct volume	Rat	Yes	30	0.80	0.64-0.89
10	TMZ	Infarct volume	Rodent	No	26	0.96	0.84-0.99
11	c-kit CSC	EF (%)	Rodent	Yes	20	0.54	0.41-0.68
12	Rat BMSC	Infarct volume	Rat	No	25	0.33	0.24-0.36

Table A: Descriptors and selection criteria for 12 additional replicate study pools. We searched the CAMARADES database for all drugs or treatments for which we found more than 25 contrasts for the same outcome measure. Outcome measures that were collective terms for various measures or tests (spec. ‘neuro-behavioural score’, ‘memory’, and ‘learning’) were not considered. Contrasts where one of the following data was missing were excluded: sample size, mean outcome, and standard deviation for control and treatment group. Studies done with non-rodent species were excluded. If the majority of studies were done with a single species, only studies with the predominating species were included (indicated by “Mouse” and “Rat” in the column *Species*), otherwise all species were included (“Rodent” in column *Species*). If species were excluded this is indicated in the column ‘restrict’. From all contrasts which stem from the same publication only one was selected following the following selection rules: 1. The contrast with the largest overall sample size was selected. 2. If more than one study had shared maximum sample size, the study with the larger sample size in the treatment group was selected. 3. If more than one study shared the maximum number of subjects in the treatment group, one study was selected randomly using a random number generator. Only treatments where 20 or more contrasts remained, after applying the exclusion criteria, entered into the final study pool. N: final number of studies after application of inclusion/exclusion criteria. For each study power was estimated for two-sided mean difference tests; expected effect size and standard deviation were based on mean values for each intervention, sample sizes as reported in the studies. Median power and interquartile range are given for each intervention. A full list with the CAMARADES identifiers of the included studies is given in the supplementary data set ‘S1_Data.csv’.

	Intervention	N	ES	S.E.	z	p	CI_L	CI_U	Q	$p(Q)$	dev	τ^2	I^2	H^2
0	Hypothermia	50	0.48	0.037	12.99	<0.0001	0.41	0.55	667.5	<0.0001	5.86	0.05	91.4	11.56
1	tPA	57	0.20	0.050	3.40	<0.0001	0.10	0.29	276.6	<0.0001	54.77	0.10	84.8	6.56
2	Trastuzumab	58	0.46	0.046	10.05	<0.0001	0.37	0.55	1071.9	<0.0001	46.30	0.09	94.6	18.37
3	FK 506	31	0.39	0.038	10.31	<0.0001	0.32	0.46	186.5	<0.0001	-8.21	0.03	81.9	5.52
4	Rosiglitazone 2	21	0.47	0.039	12.20	<0.0001	0.40	0.55	84.4	<0.0001	-12.22	0.02	73.0	3.70
5	IL-1RA	37	0.34	0.029	11.91	<0.0001	0.29	0.40	114.3	<0.0001	-23.17	0.01	54.5	2.20
6	Cardiosphere DC	35	-0.52	0.036	-14.50	<0.0001	-0.59	-0.45	248.5	<0.0001	-2.44	0.03	91.9	12.43
7	Estradiol	24	0.36	0.064	5.61	<0.0001	0.23	0.48	127.4	<0.0001	14.34	0.07	84.7	6.54
8	Human MSC	26	0.25	0.037	6.56	<0.0001	0.17	0.32	414.4	<0.0001	-9.93	0.03	97.2	35.91
9	MK 801	30	0.36	0.038	9.41	<0.0001	0.28	0.43	116.5	<0.0001	-6.30	0.03	80.7	5.19
10	TMZ	26	0.51	0.044	11.72	0.0109	0.43	0.60	176.3	<0.0001	-0.64	0.03	86.1	7.21
11	c-kit CSC	20	-0.30	0.028	-10.79	<0.0001	-0.36	-0.25	36.7	0.0078	-15.89	0.01	41.7	1.71
12	Rat BMSC	25	0.19	0.048	3.94	<0.0001	0.10	0.29	955.9	<0.0001	-3.34	0.05	94.6	18.41

Table B: Results of random effects meta-analyses with REML estimators using the R-package *metafor* 1.9-9. N: number of studies in the final study pool, ES: effect size estimate, S.E.: standard error of the effect size estimate, Q: Q-statistic for homogeneity of effect sizes, τ^2: between-study variance, dev: deviance, I^2: fraction of total heterogeneity divided by total variability, H^2: fraction of total variability divided by sampling variability. Meta-analyses were performed after scaling.

Supporting Text:

For estimating the true effect for a treatment we employed fixed effect meta-analyses. Here we will briefly discuss the reasoning of this choice. Historically, clinical multi-centre studies have been analysed in different ways: by simply pooling the data from different centres or by treating centre as a fixed or random variable. Pooling the data from different laboratories and performing a t-test or F-test on the pooled data is a problematic approach, because it clearly violates the assumption of independence of the data. This problem has been discussed at length [S1-S3] and the pitfalls of pseudo-replication by ignoring statistical dependencies are extensively treated in almost all textbooks on experimental design and statistics. We do not recommend this approach, yet we have to note that comparing the diagnostic odds ratios for pooled t-tests, 2-way ANOVAs and mixed-effect models, we see—for the 13 interventions analysed in this study—only marginal differences in the performance of the test methods (Fig. D). Having agreed on accounting for statistical dependencies, which arise from testing multiple animals in the same laboratory, we face the decision whether to treat lab-membership as a fixed or random factor. With respect to this question there seems to be no overall agreement [S4-S6]. We would argue that, conceptually, laboratory is clearly a random factor, as the laboratories, which participated in the multi-lab study, are a random sample from the set of all existing—or potentially existing—laboratories. Arguably, it is not a true random sample (amongst other reasons, national laws and regulations and regional research cultures create spatial correlations), but this might be an issue that cannot be resolved. More importantly, we have to note that this random factor will only have a very limited number of levels—from 2 to 4 in our simulations and perhaps, under rare conditions, up to 5 or 6 for large multi-lab studies. For practical and organizational reasons multi-lab studies in pre-clinical research with even higher numbers of participating laboratories seem rather unrealistic and we are not aware of any attempts at achieving that. With only a few levels of the random factor, the estimation of the hyper-parameters might be rather poor [S7]. Several authors suggested rules-of-thumb for a minimum number of levels for treating a factor as random. These rules-of-thumb typically suggest between 5 and 15 levels as a minimum. This is clearly more than the number of laboratories in a multi-lab study and, following this line of reasoning, one should better treat the factor 'laboratory' as a fixed factor. On the other side, Gellman and Hill [S7] have argued that, even in the extreme case of only two levels, the mixed-effect model does not perform worse than a fixed effect model and, therefore, it might even be appropriate in cases, where there are only very few levels of the random variable. Apart from this issue, there is the question how many degrees of freedom one should attribute to the random factor. While some authors suggest that this number can at least be approximated [S8-S10], others disagree and recommend forgoing the reporting of *p*-values and inferential hypothesis testing [S11]. The question of degrees of freedom is relevant because the estimation of the ratio p_{sa} requires repeated hypothesis testing. Therefore, we didn't want to dismiss this potential problem light-heartedly. However, we must again note that for the examined range of sample sizes and number of participating laboratories, inference based on random effect models leads to very similar results as inference based on fixed effect models (Fig. D), suggesting that both approaches can be equally feasible.

Supporting References:

S1. Johnson VE. Revised standards for statistical evidence. Proc. Natl. Acad. Sci. U. S. A. 2013; 110: 19313–19317.

S2. Benjamin DJ, Berger JO, Johannesson M, Nosek BA, Wagenmakers E-J, Berk R, et al. Redefine statistical significance. Nat. Hum. Behav. 2017; 1: 1.

S3. Bolker BM, Brooks ME, Clark CJ, Geange SW, Poulsen JR, Stevens MHH, White JSS. Generalized linear mixed models: A practical guide for ecology and evolution. Trends Ecol. Evol. 2009; 24: 127–135.

S4. Hector A, Bett T, Hautier Y, Isbell F, Kery M, Reich PB, et al. BUGS in the analysis of biodiversity experiments: Species richness and composition are of similar importance for grassland productivity. PLoS One. 2011; 6: e17434.

S5. Bennington CC, Thayne WV. Use and misuse of mixed model analysis of variance in ecological studies. Ecology. 2016; 75: 717–722.

S6. Merlo J, Chaix B, Ohlsson H, Beckman A, Johnell K, Hjerpe P, et al. A brief conceptual tutorial of multilevel analysis in social epidemiology: Using measures of clustering in multilevel logistic regression to investigate contextual phenomena. J. Epidemiol. Community Health. 2006; 60: 290–297.

S7. Gelman A, Hill J. Data analysis using regression and multilevel/hierarchical models. Cambridge: Cambridge University Press; 2007.

S8. Satterthwaite FE. An approximate distribution of estimates of variance components. Biometrics, 1946; 2: 110-114.

S9. Kenward MG, Roger JH. Small sample inference for fixed effects from restricted maximum likelihood. Biometrics. 1997; 53: 983–997.

S10. Welch BL. Note on some criticisms made by Sir Ronald Fisher. J. R. Stat. Soc. Ser. B. 1956; 18: 297–302.

S11. Bates D, Mächler M, Bolker BM, Walker SC. Fitting linear mixed-effects models using lme4. J. Stat. Softw. 2015; 67: 1–48.

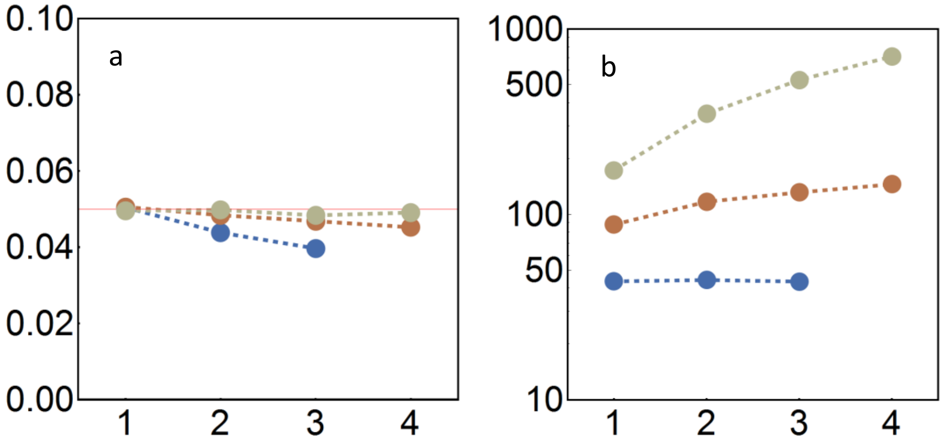

Figure B. (a) False positive rate and (b) diagnostic odds ratio (DOR) based on 10^5 simulated samples based on 50 studies on hypothermia treatment of stroke for overall sample sizes of N=12 (blue), N=24 (orange) and N=48 (grey) animal subjects and k= 1 to 4 participating laboratories. Inference was based on fixed effects 2-way ANOVA (Y= Treatment + Lab). The thin red line in panel (a) indicates the 0.05 probability threshold. The diagnostic odds ratio is the ratio of the positive likelihood ratio and the negative likelihood ratio, i.e. DOR= (true positive rate / false positive rate) / (false negative rate / true negative rate).

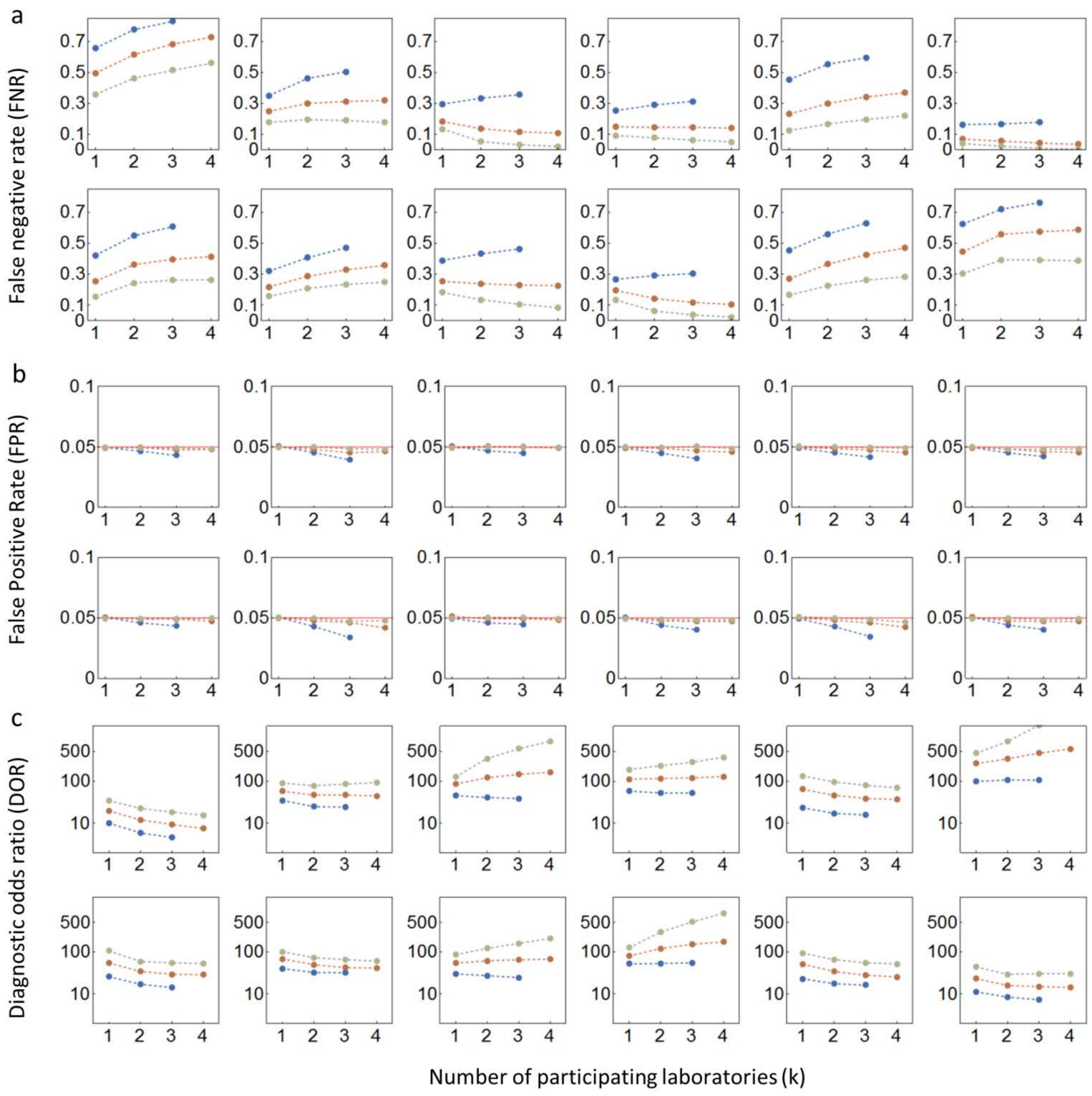

Figure C. False negative rate (a), false positive rate (b) and diagnostic odds ratio (c) for the 12 replicate data sets (from left to right: row 1: D1-D6, row 2: D7-D12), based on 10^5 simulations for overall sample sizes of N=12 (blue), N=24 (orange) and N=48 (grey) animal subjects.

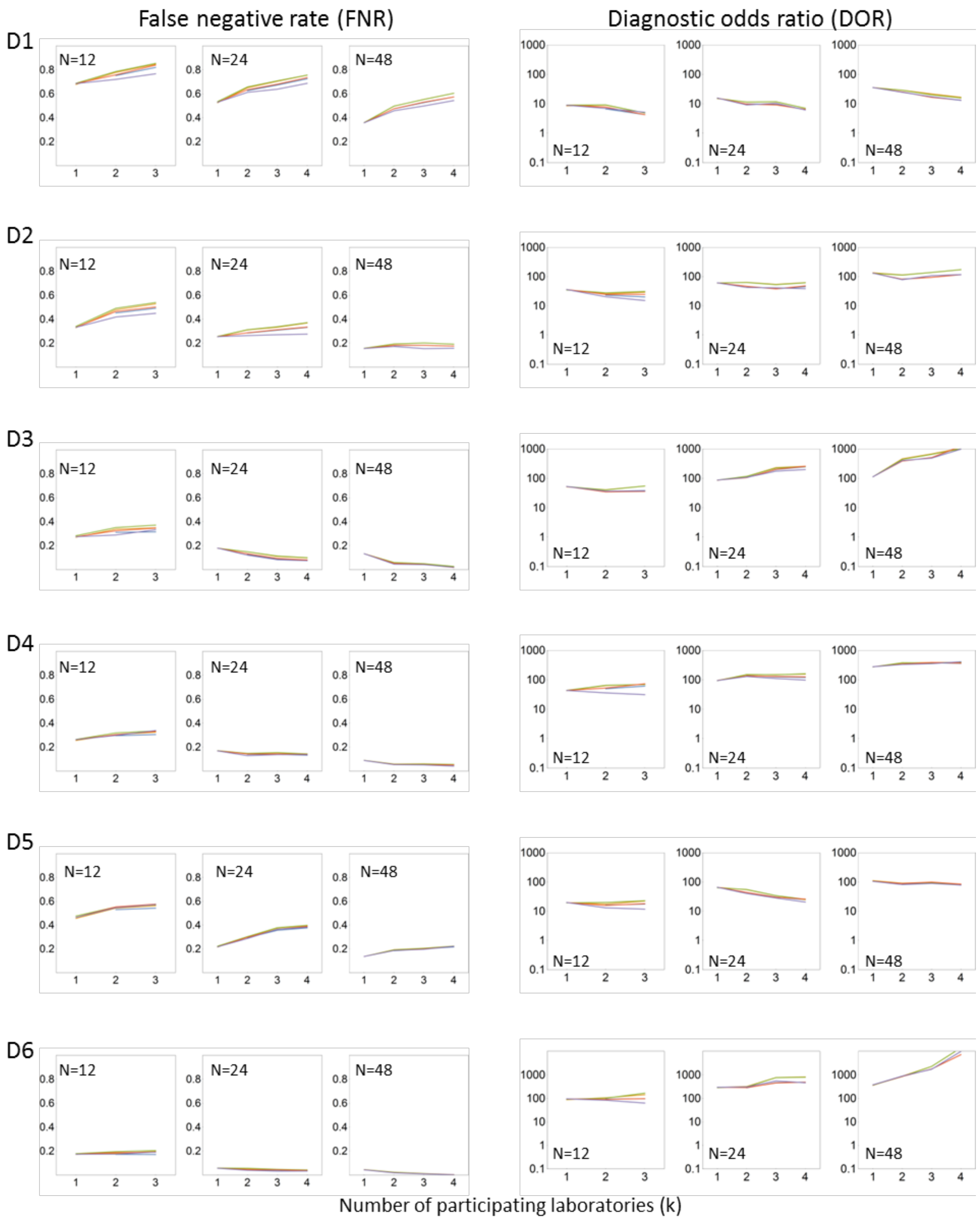

Figure D. False negative rate and diagnostic odds ratio for the 12 replicate data sets (D1-D12) with 10^3 simulations per data set. Inference based on: inclusion of zero by the parametric 95% confidence interval (yellow), t-test on pooled data (green), ANOVA with main effects treatment and laboratory only (red), ANOVA with main effects and interaction term (violet), general linear mixed model Y~treatment+(1|lab) with lab as random effect (blue). In almost all cases diagnostics based on all 5 inference techniques showed very similar behaviour, showing that the findings are not specific to the method of statistical analysis.

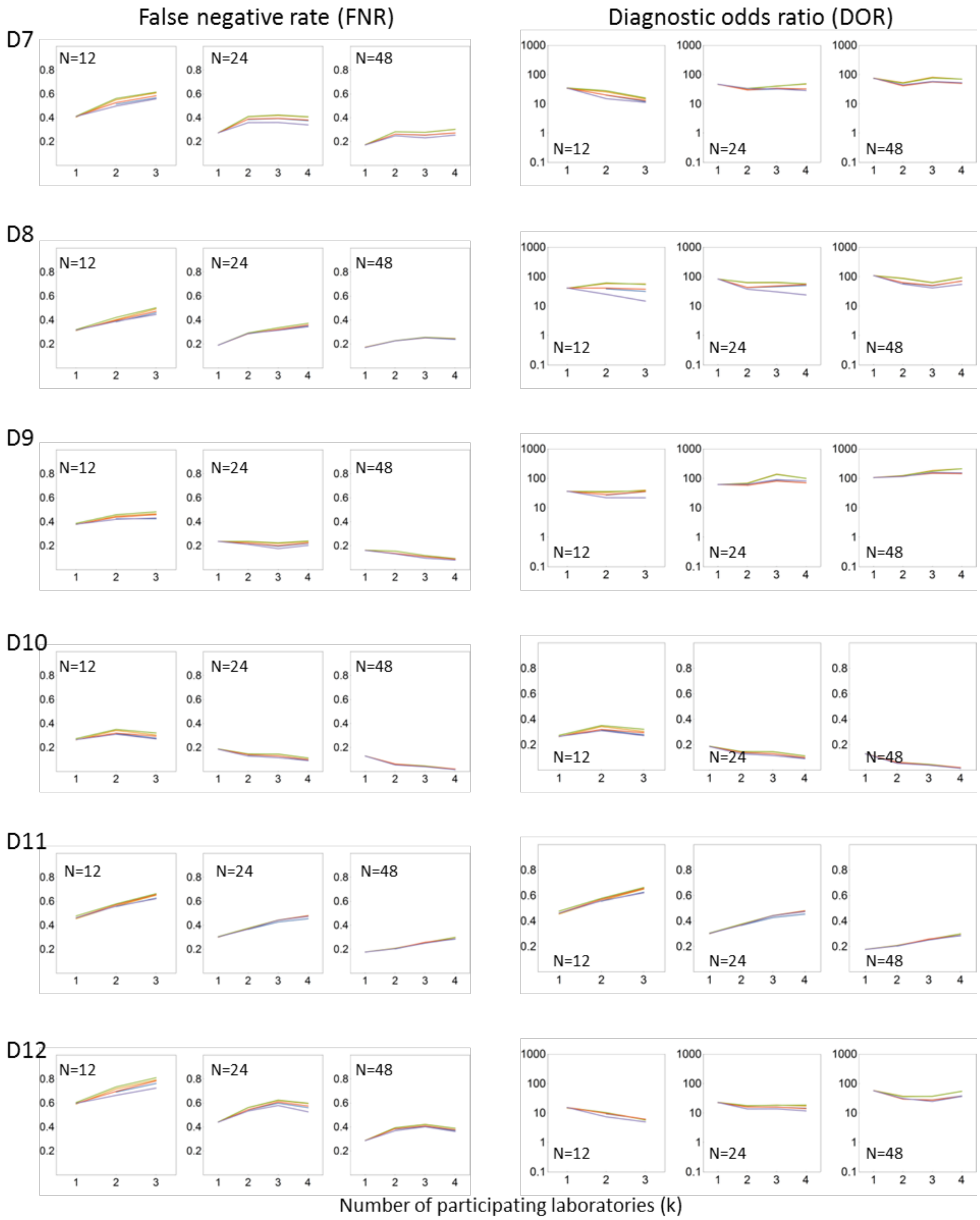

Figure D cont.

Pseudocode for simulating multi-lab studies

k = number of laboratories
n = total number of animals

```
REPEAT the following 100.000 times:
    create a list of k laboratories by sampling without replacement from the study pool
    FOR each laboratory
        control = sample n/(2k) values from a normal distribution with mean and standard deviation
            as reported for the control group and divide those values by the reported mean
            value for the control group
        treatment = sample n/(2k) values from a normal distribution with mean and standard
            deviation as reported for the treatment group and divide those values by the
            reported mean value for the control group
    ENDFOR
    perform a two-way fixed effect ANOVA on the simulated data
    pool the control values of all laboratories
    pool the treatment values of all laboratories
    calculate the means difference and 95% confidence interval for the pooled data
    test whether confidence interval includes the estimate for the means difference of the meta-analysis
ENDREPEAT
```

Mathematica Code for simulating multi-lab studies
(Effect size estimate, CI_{95}, and 2-way ANOVA)

```
getOneES[labslist_] := Module[
        {nplc, controls, treatments, se, meansdiff, lower, upper, ci, anovap},
         nplc = totalsamplesize/(k*2);        (* k is the number of labs and                          *)
                                              (* nplc is the number of animals per lab per condition  *)
         controls = Flatten[Table[RandomReal[NormalDistribution[data[[labslist[[i]], 4]],
                  data[[labslist[[i]],11]]], nplc]/data[[labslist[[i]], 4]], {i, k}]];
        treatments = Flatten[Table[RandomReal[NormalDistribution[data[[labslist[[i]], 7]],
                  data[[labslist[[i]], 12]]], nplc]/data[[labslist[[i]], 4]], {i,k}]];
                                              (* this generates values sampled from normal distributions   *)
                                              (* with parameters as reported in the original studies       *)
        se = Sqrt[(StandardDeviation[treatments]^2 + StandardDeviation[controls]^2)/(totalsamplesize/2)];
                                              (* se is the standard error for the mean difference          *)
        meansdiff = Mean[controls - treatments];   (* this is the mean difference                          *)
        anovap =                              (* the p-value of a fixed effect ANOVA                       *)
        If[k == 1,
                ANOVA[Transpose[{Join[Table[0, { totalsamplesize/2}], Table[1, { totalsamplesize/2}]],
                        Join[controls, treatments]}]][[1, 2, 1, 1, 5]],
                                              (* for the one-lab condition a one-way ANOVA is made         *)
                                              (* and the p-value is assigned to the variable anovap        *)
                ANOVA[Transpose[{Join[Table[0, {{ totalsamplesize/2}], Table[1, {{ totalsamplesize/2}]],
                        Flatten[Join[Table[Table[i, {nplc}], {i, k}], Table[Table[i, {nplc}], {i, k}]]],
                        Join[controls, treatments]}], {x, y}, {x, y}][[1, 2, 1, 1, 5]]
                                              (* for more than one lab a two-way ANOVA is made             *)
                                              (* and the p-value is assigned to the variable anovap        *)
        ];
        lower = meansdiff - zvalue*se;        (* this gives the lower 95% CI                               *)
        upper = meansdiff + zvalue*se;        (* this gives the upper 95% CI                               *)
        {truees >= lower && truees <= upper, lower > 0 || upper < 0, anovap, anovap < 0.05}
        (* this gives a list with: the first entry giving True if the true effect size lies within the 95%     *)
        (* confidence interval, and False otherwise, the second entry gives True if the 95% confidence         *)
        (* interval is not including zero and False otherwise, the third entry is the p-value estimate*)       *)
        (* from the ANOVA, and the fourth entry is True if the p-value of the ANOVA is less than 0.05          *)
        (* and False otherwise                                                                                 *)
 ]

results = Table[getOneES[RandomSample[Range[numberofstudies], k]], {100 000}];
(*this repeats the simulation 100.000 times. The number of labs (k) and the true effect size (truees)         *)
(* and the number of studies in the data matrix (numberofstudies) must be specified before executing         *)
(* the function. The data of the original studies must be provided as matrix with the observed mean          *)
(* of the control group in column 4, the mean of the treatment group in column 7, the standard               *)
(* deviation of the control group in column 11 and the standard deviation for the treatment group in         *)
(* column 12.                                                                                                *)
```

R-code for meta analyses

```
library("metafor")

data<-read.table("dataset_meta.csv", header=TRUE, sep=';')
ncontrol<-data$Number.in.Control.Group
mcontrol<-data$Reported.Mean.in.Control.Group/data$Reported.Mean.in.Control.Group
sdcontrol<-data$Calculated.SD.in.Control.Group/data$Reported.Mean.in.Control.Group
ntreatment<-data$Number.in.Treatment.Group
mtreatment<-data$Reported.Mean.in.Treatment.Group/data$Reported.Mean.in.Control.Group
sdtreatment<-data$Calculated.SD.in.Treatment.Group/data$Reported.Mean.in.Control.Group
# This block reads in observed values from data for reported sample size of the control
# group (ncontrol), reported mean of the control group (mcontrol), reported standard deviation
# for the control group (sdcontrol), reported sample size of the treatment group (ntreatment),
# reported mean of the treatment group (mtreatment), and reported standard deviation for
# the treatment group (sdtreatment).

result.meta <- rma(m1=mcontrol, m2=mtreatment,
        sd1=sdcontrol, sd2=sdtreatment, n1= ncontrol, n2=ntreatment,
        method="REML", measure="MD")            # ”MD” indicates means difference

summary(result.meta)
```
