# Supplementary material for: Reproducibility of preclinical animal research improves with heterogeneity of study samples
Source: PLoS Biol. 2018 Feb 22;16(2):e2003693. doi: 10.1371/journal.pbio.2003693 (PMC5823461; doi:10.1371/journal.pbio.2003693)
Supplement: S2 Text — (PDF) [file pbio.2003693.s002.pdf]

Initialization

```
Needs["ANOVA`"]
```

Functions

```
zvalue[n_] := Which[n == 12, 2.201, n == 24, 2.069, n == 48, 2.021]

getOneES[labslist_] := Module[{animalsperlabcondition, controls,
   treatments, se, meansdiff, lower, upper, ci, tp, anovap, ianovap},
  animalsperlabcondition = totalsamplesize / (Length[labslist] * 2);
  controls = Flatten[Table[
      RandomReal[NormalDistribution[data[[labslist[[i]], 4]], data[[labslist[[i]], 6]]],
        animalsperlabcondition] / data[[labslist[[i]], 4]], {i, Length[labslist]}]];
  treatments = Flatten[Table[RandomReal[NormalDistribution[
        data[[labslist[[i]], 5]], data[[labslist[[i]], 7]]], animalsperlabcondition] /
      data[[labslist[[i]], 4]], {i, Length[labslist]}]];
  se = Sqrt[(StandardDeviation[treatments]^2 + StandardDeviation[controls]^2) /
     (totalsamplesize / 2)];
  meansdiff = Mean[controls - treatments];
  tp = Quiet[TTest[{controls, treatments}]];
  anovap =
   If[Length[labslist] == 1, ANOVA[Transpose[{Join[Table[0, {Length[controls]}],
          Table[1, {Length[treatments]}]], Join[controls, treatments]}]][[
     1, 2, 1, 1, 5]], ANOVA[Transpose[{Join[Table[0, {Length[controls]}],
          Table[1, {Length[treatments]}]],
         Flatten[Join[Table[Table[i, {animalsperlabcondition}], {i, nlabs}],
           Table[Table[i, {animalsperlabcondition}], {i, nlabs}]]],
         Join[controls, treatments]}], {x, y}, {x, y}][[1, 2, 1, 1, 5]]];
  ianovap = If[Length[labslist] == 1, ANOVA[Transpose[
        {Join[Table[0, {Length[controls]}], Table[1, {Length[treatments]}]],
         Join[controls, treatments]}]][[1, 2, 1, 1, 5]], ANOVA[Transpose[
        {Join[Table[0, {Length[controls]}], Table[1, {Length[treatments]}]],
         Flatten[Join[Table[Table[i, {animalsperlabcondition}], {i, nlabs}],
           Table[Table[i, {animalsperlabcondition}], {i, nlabs}]]],
         Join[controls, treatments]}], {x, y, x y}, {x, y}][[1, 2, 1, 1, 5]]];
  lower = meansdiff - zvalue[totalsamplesize] * se;
  upper = meansdiff + zvalue[totalsamplesize] * se;
  ci = upper - lower;
  {truees ≥ lower && truees ≤ upper, lower > 0 || upper < 0, tp, tp < 0.05,
   anovap, anovap < 0.05, ianovap, ianovap < 0.05, controls, treatments}
  (*1. capture, 2. sig CI method, 3. p (t-test), 4. sig (t-test),
  5. p (x+y ANOVA), 6. sig (x+y ANOVA), 7. p (x*y ANOVA),
  8. sig (x*y ANOVA), 9. controls, 10. treatments*)
 ]
```

```
getNoDifferenceES[labslist_] :=
 Module[{animalsperlabcondition, controls, treatments,
   se, meansdiff, lower, upper, ci, tp, anovap, ianovap},
  animalsperlabcondition = totalsamplesize / (Length[labslist] * 2);
  controls = Flatten[Table[
     RandomReal[NormalDistribution[(data[[labslist[[i]], 5]] + data[[labslist[[i]], 4]]) /
          2, (data[[labslist[[i]], 6]] + data[[labslist[[i]], 7]]) / 2],
       animalsperlabcondition] / data[[labslist[[i]], 4]], {i, Length[labslist]}]];
  treatments = Flatten[Table[RandomReal[NormalDistribution[
        (data[[labslist[[i]], 5]] + data[[labslist[[i]], 4]]) / 2,
        (data[[labslist[[i]], 6]] + data[[labslist[[i]], 7]]) / 2],
       animalsperlabcondition] / data[[labslist[[i]], 4]], {i, Length[labslist]}]];
  se = Sqrt[(StandardDeviation[treatments] ^2 + StandardDeviation[controls] ^2) /
     (totalsamplesize / 2)];
  meansdiff = Mean[controls - treatments];
  tp = Quiet[TTest[{controls, treatments}]];
  anovap =
   If[Length[labslist] == 1, ANOVA[Transpose[{Join[Table[0, {Length[controls]}],
          Table[1, {Length[treatments]}]], Join[controls, treatments]}]][[
     1, 2, 1, 1, 5]], ANOVA[Transpose[{Join[Table[0, {Length[controls]}],
          Table[1, {Length[treatments]}]],
         Flatten[Join[Table[Table[i, {animalsperlabcondition}], {i, nlabs}],
           Table[Table[i, {animalsperlabcondition}], {i, nlabs}]]],
         Join[controls, treatments]}], {x, y}, {x, y}][[1, 2, 1, 1, 5]]];
  ianovap = If[Length[labslist] == 1, ANOVA[Transpose[
        {Join[Table[0, {Length[controls]}], Table[1, {Length[treatments]}]],
         Join[controls, treatments]}]][[1, 2, 1, 1, 5]], ANOVA[Transpose[
        {Join[Table[0, {Length[controls]}], Table[1, {Length[treatments]}]],
         Flatten[Join[Table[Table[i, {animalsperlabcondition}], {i, nlabs}],
           Table[Table[i, {animalsperlabcondition}], {i, nlabs}]]],
         Join[controls, treatments]}], {x, y, x y}, {x, y}][[1, 2, 1, 1, 5]]];
  lower = meansdiff - zvalue[totalsamplesize] * se;
  upper = meansdiff + zvalue[totalsamplesize] * se;
  ci = upper - lower;
  {truees ≥ lower && truees ≤ upper, lower > 0 || upper < 0, tp, tp < 0.05,
   anovap, anovap < 0.05, ianovap, ianovap < 0.05, controls, treatments}
  (*1. capture, 2. sig CI method, 3. p (t-test), 4. sig (t-test),
  5. p (x+y ANOVA), 6. sig (x+y ANOVA), 7. p (x*y ANOVA),
  8. sig (x*y ANOVA), 9. controls, 10. treatments*)
 ]
```

```
getCI[{controls_, treatments_}] := Module[{se, meansdiff, lower, upper, ci},
  se = Sqrt[(StandardDeviation[treatments]^2 + StandardDeviation[controls]^2) /
     (totalsamplesize / 2)];
  meansdiff = Mean[controls - treatments];
  lower = meansdiff - zvalue[totalsamplesize] * se;
  upper = meansdiff + zvalue[totalsamplesize] * se;
  ci = upper - lower;
  ci
 ]

dor[{tpr_, fpr_}] := (tpr / fpr) / ((1 - tpr) / (1 - fpr))

summary[mat_] := {N[Count[mat[[All, 1]], True] / Length[mat]],
  N[Count[mat[[All, 2]], True] / Length[mat]],
  Mean[mat[[All, 3]]], N[Count[mat[[All, 4]], True] / Length[mat]],
  Mean[mat[[All, 5]]], N[Count[mat[[All, 6]], True] / Length[mat]],
  Mean[mat[[All, 7]]], N[Count[mat[[All, 8]], True] / Length[mat]]}

getParam[labslist_] := Module[{animalsperlabcondition,
   controls, treatments, se, meansdiff, lower, upper, ci},
  animalsperlabcondition = totalsamplesize / (Length[labslist] * 2);
  controls = Flatten[Table[
     RandomReal[NormalDistribution[data[[labslist[[i]], 4]], data[[labslist[[i]], 6]]],
       animalsperlabcondition] / data[[labslist[[i]], 4]], {i, Length[labslist]}]];
  treatments = Flatten[Table[RandomReal[NormalDistribution[
        data[[labslist[[i]], 5]], data[[labslist[[i]], 7]]], animalsperlabcondition] /
      data[[labslist[[i]], 4]], {i, Length[labslist]}]];
  se = Sqrt[(StandardDeviation[treatments]^2 + StandardDeviation[controls]^2) /
     (totalsamplesize / 2)];
  meansdiff = Mean[controls - treatments];
  lower = meansdiff - zvalue[totalsamplesize] * se;
  upper = meansdiff + zvalue[totalsamplesize] * se;
  ci = upper - lower;
  {Mean[controls], Mean[treatments],
   StandardDeviation[controls], StandardDeviation[treatments],
   Round[totalsamplesize / 2], Round[totalsamplesize / 2]}
 ]

calcMeansDiff[{meanc_, meant_, sdc_, sdt_, nc_, nt_}] := {meanc - meant, Null,
  (meanc - meant) - Sqrt[sdc^2 + sdt^2], (meanc - meant) + Sqrt[sdc^2 + sdt^2]}

makeLineS[simdata_, i_] := Line[{{simdata[[i, -2]], i}, {simdata[[i, -1]], i}}]

makePointS[simdata_, i_] := Point[{simdata[[i, -4]], i}]
```

```
figcpF[df1data_] := Show[ListLinePlot[df1data,
    PlotStyle → {Directive[Dashed, Thickness[0.01], RGBColor[0.27, 0.42, 0.67]],
      Directive[Dashed, Thickness[0.01], RGBColor[0.82, 0.45, 0.29]],
      Directive[Dashed, Thickness[0.01], RGBColor[0.60, 0.6, 0.6]]},
    Frame → True, FrameStyle → Thickness[0.005],
    FrameTicks → {{{0, 0.2, 0.4, 0.6, 0.8}, None}, {{1, 2, 3, 4}, None}},
    FrameTicksStyle → Directive[Black, 28],
    PlotRange → {{0.8, 4.2}, {0, 1}}, AspectRatio → 1], ListPlot[df1data,
    PlotStyle → {Directive[PointSize[0.05], RGBColor[0.27, 0.42, 0.67]],
      Directive[PointSize[0.05], RGBColor[0.72, 0.45, 0.29]],
      Directive[PointSize[0.05], RGBColor[0.70, 0.7, 0.56]]}]];

figfnrF[df2data_] := Show[ListLinePlot[df2data,
    PlotStyle → {Directive[Dashed, Thickness[0.01], RGBColor[0.27, 0.42, 0.67]],
      Directive[Dashed, Thickness[0.01], RGBColor[0.82, 0.45, 0.29]],
      Directive[Dashed, Thickness[0.01], RGBColor[0.60, 0.6, 0.6]]},
    Frame → True, FrameStyle → Thickness[0.005],
    FrameTicks → {{{0, 0.1, 0.3, 0.5, 0.7}, None}, {{1, 2, 3, 4}, None}},
    FrameTicksStyle → Directive[Black, 28],
    PlotRange → {{0.8, 4.2}, {0, 0.85}}, AspectRatio → 1], ListPlot[df2data,
    PlotStyle → {Directive[PointSize[0.05], RGBColor[0.27, 0.42, 0.67]],
      Directive[PointSize[0.05], RGBColor[0.72, 0.45, 0.29]],
      Directive[PointSize[0.05], RGBColor[0.70, 0.7, 0.56]]}]];

figdorF[df3data_] := Show[ListLinePlot[df3data, ScalingFunctions → "Log",
    PlotStyle → {Directive[Dashed, Thickness[0.01], RGBColor[0.27, 0.42, 0.67]],
      Directive[Dashed, Thickness[0.01], RGBColor[0.82, 0.45, 0.29]],
      Directive[Dashed, Thickness[0.01], RGBColor[0.60, 0.6, 0.6]]},
    Frame → True, FrameStyle → Thickness[0.005],
    FrameTicks → {{{10, 100, 500}, None}, {{1, 2, 3, 4}, None}},
    FrameTicksStyle → Directive[Black, 28],
    PlotRange → {{0.5, 4.5}, {2, 2000}}, AspectRatio → 1],
   ListPlot[df3data, ScalingFunctions → "Log",
    PlotStyle → {Directive[PointSize[0.05], RGBColor[0.27, 0.42, 0.67]],
      Directive[PointSize[0.05], RGBColor[0.72, 0.45, 0.29]],
      Directive[PointSize[0.05], RGBColor[0.70, 0.7, 0.56]]}]];

cs[input_] := Module[{a},
  a = Count[Table[input[[i, 1]] && input[[i, 6]], {i, Length[input]}], True];
  N[a / Length[input]]
 ](*gives the proportion of correct outcomes of all studies*)
```

```
power[{x1_, x2_, s1_, s2_, n1_, n2_}] := Module[{d, criticalt, delta, meansdiff},
  meansdiff = Abs[x1 - x2];
  d = meansdiff / Sqrt[(s1^2 + s2^2) / 2];
  criticalt =
   InverseSurvivalFunction[StudentTDistribution[0, 1, (n1 + n2 - 2)], 0.025];
  delta = d Sqrt[(n1 n2) / (n1 + n2)];
  1 - NIntegrate[PDF[StudentTDistribution[delta, 1, (n1 + n2 - 2)], x],
    {x, -criticalt, criticalt}]
 ]
```

1. Hypothermia Data Set Meta Analysis

The true effect size was estimated using a random effects meta analysis (with REML estimator) in the R-package *metafor* (file MultilabR04.R). The weighted means difference is **0.4781**

```
truees = 0.4781
```

2. Simulation

Simulation

```
nsims = 100 000;
```

```
totalsamplesize = 12; (*This is the total number of animals
 in the study. To be repeated with 24 and 48 accordingly*)
```

```
nlabs = 1; (*nlabs is the number of participating laboratories. To be
 repeated with 2, 3, and for total sample sizes of 24 and 48 also 4*)
res = Table[getOneES[RandomSample[Range[50], nlabs]], {nsims}];
```

```
{Count[res[[All, 1]], True] / (nsims / 100.),
 Count[res[[All, 2]], True] / (nsims / 100.), Count[res[[All, 4]], True] / (nsims / 100.),
 Count[res[[All, 6]], True] / (nsims / 100.), Count[res[[All, 8]], True] / (nsims / 100.)}
```

This gives 1. capture rate, 2. TPR CI method, 3. TPR t-test, 4. TPR main effect ANOVA, 5. TPR full ANOVA

```
contr = res[[All, 9]];
Export[StringJoin["ml4n",
   ToString[totalsamplesize], "l", ToString[nlabs], "c.csv"], contr];
treatm = res[[All, 10]];
Export[StringJoin["ml4n",
   ToString[totalsamplesize], "l", ToString[nlabs], "t.csv"], treatm];
result = res[[All, 1 ;; 8]];
Save[StringJoin["ml4n",
   ToString[totalsamplesize], "l", ToString[nlabs], "res"], result];
```

```
resnd = Table[getNoDifferenceES[RandomSample[Range[50], nlabs]], {nsims}];
```

```
{Count[resnd[[All, 1]], True] / (nsims / 100.),
 Count[resnd[[All, 2]], True] / (nsims / 100.),
 Count[resnd[[All, 4]], True] / (nsims / 100.),
 Count[resnd[[All, 6]], True] / (nsims / 100.),
 Count[resnd[[All, 8]], True] / (nsims / 100.)}
```

This gives 1. capture rate, 2. FPR CI method, 3. FPR t-test, 4. FPR main effect ANOVA, 5. FPR full ANOVA

```
contrnd = resnd[[All, 9]];
Export[StringJoin["ml4ndn",
   ToString[totalsamplesize], "l", ToString[nlabs], "c.csv"], contrnd];
treatmnd = resnd[[All, 10]];
Export[StringJoin["ml4ndn",
   ToString[totalsamplesize], "l", ToString[nlabs], "t.csv"], treatmnd];
resultnd = resnd[[All, 1 ;; 8]];
Save[StringJoin["ml4ndn",
   ToString[totalsamplesize], "l", ToString[nlabs], "res"], resultnd];
```

CIs

```
totalsamplesize = 12;
(*To be repeated for N= 24 and 48 and nlabs=2, 3, 4 accordingly*)
nlabs = 1;
control = Import[StringJoin["ml4n",
    ToString[totalsamplesize], "l", ToString[nlabs], "c.csv"]];
treatment = Import[StringJoin["ml4n", ToString[totalsamplesize],
    "l", ToString[nlabs], "t.csv"]];
mat = Transpose[{control, treatment}];
ciwn12l1 = Mean[getCI /@ mat];
```

Figure 2B - 2D

```
r12l1 = summary[Get[StringJoin["ml4n", ToString[12], "l", ToString[1], "res"]]];
r12l2 = summary[Get[StringJoin["ml4n", ToString[12], "l", ToString[2], "res"]]];
r12l3 = summary[Get[StringJoin["ml4n", ToString[12], "l", ToString[3], "res"]]];
r24l1 = summary[Get[StringJoin["ml4n", ToString[24], "l", ToString[1], "res"]]];
r24l2 = summary[Get[StringJoin["ml4n", ToString[24], "l", ToString[2], "res"]]];
r24l3 = summary[Get[StringJoin["ml4n", ToString[24], "l", ToString[3], "res"]]];
r24l4 = summary[Get[StringJoin["ml4n", ToString[24], "l", ToString[4], "res"]]];
r48l1 = summary[Get[StringJoin["ml4n", ToString[48], "l", ToString[1], "res"]]];
r48l2 = summary[Get[StringJoin["ml4n", ToString[48], "l", ToString[2], "res"]]];
r48l3 = summary[Get[StringJoin["ml4n", ToString[48], "l", ToString[3], "res"]]];
r48l4 = summary[Get[StringJoin["ml4n", ToString[48], "l", ToString[4], "res"]]];
```

```
cp12 = {r12l1[[1]], r12l2[[1]], r12l3[[1]]};
cp24 = {r24l1[[1]], r24l2[[1]], r24l3[[1]], r24l4[[1]]};
cp48 = {r48l1[[1]], r48l2[[1]], r48l3[[1]], r48l4[[1]]};

(*This gives the coverage probability for N=12, 24 and 48 and 1 to 4 labs *)

wci12 = {ciwn12l1, ciwn12l2, ciwn12l3};
wci24 = {ciwn24l1, ciwn24l2, ciwn24l3, ciwn24l4};
wci48 = {ciwn48l1, ciwn48l2, ciwn48l3, ciwn48l4};

(*This gives the widths of the confidence intervals for N=12,
24 and 48 and 1 to 4 labs *)

fnr12 = 1 - {r12l1[[6]], r12l2[[6]], r12l3[[6]]};
fnr24 = 1 - {r24l1[[6]], r24l2[[6]], r24l3[[6]], r24l4[[6]]};
fnr48 = 1 - {r48l1[[6]], r48l2[[6]], r48l3[[6]], r48l4[[6]]};

(*This gives the false negative rate for N=12, 24 and 48 and 1 to 4 labs *)
```

Supplementary S2

```
nr12l1 =
  summary[Get[StringJoin["ml4ndn", ToString[12], "l", ToString[1], "res"]]];
nr12l2 = summary[Get[StringJoin["ml4ndn",
     ToString[12], "l", ToString[2], "res"]]];
nr12l3 = summary[Get[StringJoin["ml4ndn", ToString[12],
     "l", ToString[3], "res"]]];
nr24l1 = summary[Get[StringJoin["ml4ndn", ToString[24],
     "l", ToString[1], "res"]]];
nr24l2 = summary[Get[StringJoin["ml4ndn", ToString[24],
     "l", ToString[2], "res"]]];
nr24l3 = summary[Get[StringJoin["ml4ndn", ToString[24],
     "l", ToString[3], "res"]]];
nr24l4 = summary[Get[StringJoin["ml4ndn", ToString[24],
     "l", ToString[4], "res"]]];
nr48l1 = summary[Get[StringJoin["ml4ndn", ToString[48],
     "l", ToString[1], "res"]]];
nr48l2 = summary[Get[StringJoin["ml4ndn", ToString[48],
     "l", ToString[2], "res"]]];
nr48l3 = summary[Get[StringJoin["ml4ndn", ToString[48],
     "l", ToString[3], "res"]]];
nr48l4 = summary[Get[StringJoin["ml4ndn", ToString[48],
     "l", ToString[4], "res"]]];

fpr12 = {nr12l1[[6]], nr12l2[[6]], nr12l3[[6]]};
fpr24 = {nr24l1[[6]], nr24l2[[6]], nr24l3[[6]], nr24l4[[6]]};
fpr48 = {nr48l1[[6]], nr48l2[[6]], nr48l3[[6]], nr48l4[[6]]};

fdor12 = {{r12l1[[6]], nr12l1[[6]]}, {r12l2[[6]], nr12l2[[6]]}, {r12l3[[6]], nr12l3[[6]]}}
```

```
fdor24 = {{r24l1[[6]], nr24l1[[6]]}, {r24l2[[6]], nr24l2[[6]]},
   {r24l3[[6]], nr24l3[[6]]}, {r24l4[[6]], nr24l4[[6]]}}
```

```
fdor48 = {{r48l1[[6]], nr48l1[[6]]}, {r48l2[[6]], nr48l2[[6]]},
   {r48l3[[6]], nr48l3[[6]]}, {r48l4[[6]], nr48l4[[6]]}}
```

```
{dor /@ fdor12, dor /@ fdor24, dor /@ fdor48}
```

PSA

```
d12l1 = Get[StringJoin["ml4n", ToString[12], "l", ToString[1], "res"]];
d12l2 = Get[StringJoin["ml4n", ToString[12], "l", ToString[2], "res"]];
d12l3 = Get[StringJoin["ml4n", ToString[12], "l", ToString[3], "res"]];
d24l1 = Get[StringJoin["ml4n", ToString[24], "l", ToString[1], "res"]];
d24l2 = Get[StringJoin["ml4n", ToString[24], "l", ToString[2], "res"]];
d24l3 = Get[StringJoin["ml4n", ToString[24], "l", ToString[3], "res"]];
d24l4 = Get[StringJoin["ml4n", ToString[24], "l", ToString[4], "res"]];
d48l1 = Get[StringJoin["ml4n", ToString[48], "l", ToString[1], "res"]];
d48l2 = Get[StringJoin["ml4n", ToString[48], "l", ToString[2], "res"]];
d48l3 = Get[StringJoin["ml4n", ToString[48], "l", ToString[3], "res"]];
d48l4 = Get[StringJoin["ml4n", ToString[48], "l", ToString[4], "res"]];
```

```
hypocs12 = cs /@ {d12l1, d12l2, d12l3}
```

```
hypocs24 = cs /@ {d24l1, d24l2, d24l3, d24l4}
```

```
hypocs48 = cs /@ {d48l1, d48l2, d48l3, d48l4}
```

3. Comparing Inference Techniques

Systematic comparison FNR

```
row = {"12k2", "12k3", "24k1", "24k2", "24k3", "48k2", "48k3", "48k4"};
glmm2 = Transpose[Prepend[Table[1 - Import[
        StringJoin["F:/Paper Multilab/Analysis 2/GLMMRES01/glmmoutcomesD",
         ToString[i], "c.csv"]][[2 ;; 9, 6]], {i, 12}], row]];
```

Study 1

```
sn = 1;
d = ToString[sn];
n12l1 = Get[StringJoin["d", d, "ml4n12l1res"]];
n12l2 = Get[StringJoin["d", d, "ml4n12l2res"]];
n12l3 = Get[StringJoin["d", d, "ml4n12l3res"]];
d1n12m2 = Prepend[glmm2[[1 ;; 2, sn + 1]], Null ];
d1n12ci = {Count[n12l1[[All, 2]], False],
    Count[n12l2[[All, 2]], False], Count[n12l3[[All, 2]], False]} / 1000;
d1n12t = {Count[n12l1[[All, 4]], False], Count[n12l2[[All, 4]], False],
```

```
     Count[n12l3[[All, 4]], False]} / 1000;
d1n12anova = {Count[n12l1[[All, 6]], False], Count[n12l2[[All, 6]], False],
     Count[n12l3[[All, 6]], False]} / 1000;
d1n12anovax = {Count[n12l1[[All, 8]], False], Count[n12l2[[All, 8]], False],
     Count[n12l3[[All, 8]], False]} / 1000;
p12 = ListLinePlot[{d1n12m2, d1n12ci, d1n12t, d1n12anova, d1n12anovax},
    Frame -> True, AspectRatio → 1, PlotRange → {{0.5, 3.5}, {0, 1}},
    FrameTicks → {{{0.2, 0.4, 0.6, 0.8}, None}, {{1, 2, 3, 4}, None}},
    FrameTicksStyle → Directive[Black, 20]];
n24l1 = Get[StringJoin["d", d, "ml4n24l1res"]];
n24l2 = Get[StringJoin["d", d, "ml4n24l2res"]];
n24l3 = Get[StringJoin["d", d, "ml4n24l3res"]];
n24l4 = Get[StringJoin["d", d, "ml4n24l4res"]];
d1n24m2 = Prepend[glmm2[[3 ;; 5, sn + 1]], Null ];
d1n24ci = {Count[n24l1[[All, 2]], False], Count[n24l2[[All, 2]], False],
     Count[n24l3[[All, 2]], False], Count[n24l4[[All, 2]], False]} / 1000;
d1n24t = {Count[n24l1[[All, 4]], False], Count[n24l2[[All, 4]], False],
     Count[n24l3[[All, 4]], False], Count[n24l4[[All, 4]], False]} / 1000;
d1n24anova = {Count[n24l1[[All, 6]], False], Count[n24l2[[All, 6]], False],
     Count[n24l3[[All, 6]], False], Count[n24l4[[All, 6]], False]} / 1000;
d1n24anovax = {Count[n24l1[[All, 8]], False], Count[n24l2[[All, 8]], False],
     Count[n24l3[[All, 8]], False], Count[n24l4[[All, 8]], False]} / 1000;
p24 = ListLinePlot[{d1n24m2, d1n24ci, d1n24t, d1n24anova, d1n24anovax},
    Frame -> True, AspectRatio → 1, PlotRange → {{0.5, 4.5}, {0, 1}},
    FrameTicks → {{{0.2, 0.4, 0.6, 0.8}, None}, {{1, 2, 3, 4}, None}},
    FrameTicksStyle → Directive[Black, 20]];
n48l1 = Get[StringJoin["d", d, "ml4n48l1res"]];
n48l2 = Get[StringJoin["d", d, "ml4n48l2res"]];
n48l3 = Get[StringJoin["d", d, "ml4n48l3res"]];
n48l4 = Get[StringJoin["d", d, "ml4n48l4res"]];
d1n48m2 = Prepend[glmm2[[6 ;; 8, sn + 1]], Null ];
d1n48ci = {Count[n48l1[[All, 2]], False], Count[n48l2[[All, 2]], False],
     Count[n48l3[[All, 2]], False], Count[n48l4[[All, 2]], False]} / 1000;
d1n48t = {Count[n48l1[[All, 4]], False], Count[n48l2[[All, 4]], False],
     Count[n48l3[[All, 4]], False], Count[n48l4[[All, 4]], False]} / 1000;
d1n48anova = {Count[n48l1[[All, 6]], False], Count[n48l2[[All, 6]], False],
     Count[n48l3[[All, 6]], False], Count[n48l4[[All, 6]], False]} / 1000;
d1n48anovax = {Count[n48l1[[All, 8]], False], Count[n48l2[[All, 8]], False],
     Count[n48l3[[All, 8]], False], Count[n48l4[[All, 8]], False]} / 1000;
p48 = ListLinePlot[{d1n48m2, d1n48ci, d1n48t, d1n48anova, d1n48anovax},
    Frame -> True, AspectRatio → 1, PlotRange → {{0.5, 4.5}, {0, 1}},
    FrameTicks → {{{0.2, 0.4, 0.6, 0.8}, None}, {{1, 2, 3, 4}, None}},
    FrameTicksStyle → Directive[Black, 20]];
cg1 = GraphicsGrid[{{p12, p24, p48}}, ImageSize → 800]
```

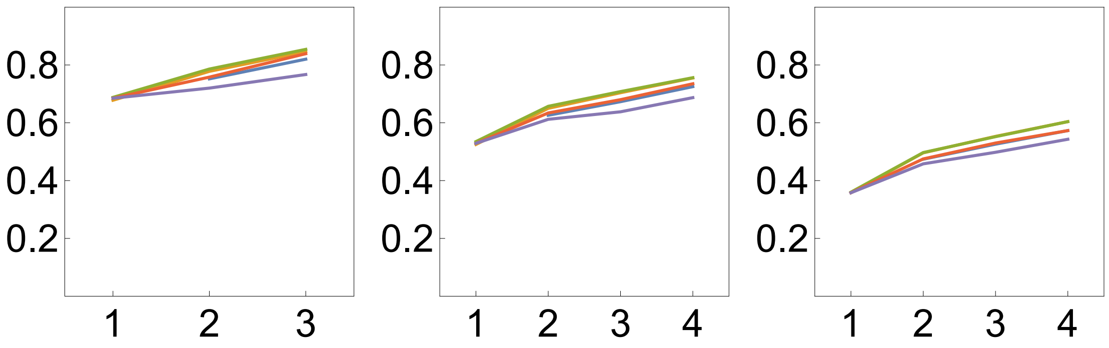

Lines: blue: GLMM: treatment+(1 | lab), violet: ANOVA x+y+xy, red: ANOVA x+y, green: pooled t-test, yellow: CI95%

Systematic Comparison DOR

```
row = {"12k2", "12k3", "24k1", "24k2", "24k3", "48k2", "48k3", "48k4"};
glmm2 = Transpose[Prepend[Table[1 - Import[
        StringJoin["F:/Paper Multilab/Analysis 2/GLMMRES01/glmmoutcomesD",
         ToString[i], "c.csv"]][[2 ;; 9, 6]], {i, 12}], row]];
```

```
row = {"12k2", "12k3", "24k1", "24k2", "24k3", "48k2", "48k3", "48k4"};
glmm2n = Transpose[Prepend[Table[1 - Import[
        StringJoin["F:/Paper Multilab/Analysis 2/GLMMRES01/glmmoutcomesD",
         ToString[i], "n.csv"]][[2 ;; 9, 6]], {i, 12}], row]];
```

Study 1

```
sn = 1;
d = ToString[sn];
n12l1 = Get[StringJoin["d", d, "ml4n12l1res"]];
n12l2 = Get[StringJoin["d", d, "ml4n12l2res"]];
n12l3 = Get[StringJoin["d", d, "ml4n12l3res"]];
ndn12l1 = Get[StringJoin["d", d, "ml4ndn12l1res"]];
ndn12l2 = Get[StringJoin["d", d, "ml4ndn12l2res"]];
ndn12l3 = Get[StringJoin["d", d, "ml4ndn12l3res"]];

d1n12m2 = Prepend[1 - glmm2[[1 ;; 2, sn + 1]], Null ];
d1n12ci = 1 - {Count[n12l1[[All, 2]], False],
     Count[n12l2[[All, 2]], False], Count[n12l3[[All, 2]], False]} / 1000;
d1n12t = 1 - {Count[n12l1[[All, 4]], False], Count[n12l2[[All, 4]], False],
     Count[n12l3[[All, 4]], False]} / 1000;
d1n12anova = 1 - {Count[n12l1[[All, 6]], False], Count[n12l2[[All, 6]], False],
     Count[n12l3[[All, 6]], False]} / 1000;
d1n12anovax = 1 - {Count[n12l1[[All, 8]], False], Count[n12l2[[All, 8]], False],
     Count[n12l3[[All, 8]], False]} / 1000;

ndd1n12m2 = Prepend[1 - glmm2n[[1 ;; 2, sn + 1]], Null ];
```

```
nddln12ci = 1 - {Count[ndn12l1[[All, 2]], False],
      Count[ndn12l2[[All, 2]], False], Count[ndn12l3[[All, 2]], False]} / 1000;
nddln12t = 1 - {Count[ndn12l1[[All, 4]], False], Count[ndn12l2[[All, 4]], False],
      Count[ndn12l3[[All, 4]], False]} / 1000;
nddln12anova = 1 - {Count[ndn12l1[[All, 6]], False], Count[ndn12l2[[All, 6]], False],
      Count[ndn12l3[[All, 6]], False]} / 1000;
nddln12anovax = 1 - {Count[ndn12l1[[All, 8]], False],
      Count[ndn12l2[[All, 8]], False], Count[ndn12l3[[All, 8]], False]} / 1000;

p12 =
  ListLinePlot[{dor[{dln12m2, nddln12m2}] //. 1 → Null, dor[{dln12ci, nddln12ci}],
     dor[{dln12t, nddln12t}], dor[{dln12anova, nddln12anova}],
     dor[{dln12anovax, nddln12anovax}]}, ScalingFunctions → "Log",
    Frame -> True, AspectRatio → 1, PlotRange → {{0.5, 3.5}, {0.1, 1000}},
    FrameTicks → {{{0.1, 0.1, 1, 10, 100, 1000}, None}, {{1, 2, 3, 4}, None}},
    FrameTicksStyle → Directive[Black, 20]];
n24l1 = Get[StringJoin["d", d, "ml4n24l1res"]];
n24l2 = Get[StringJoin["d", d, "ml4n24l2res"]];
n24l3 = Get[StringJoin["d", d, "ml4n24l3res"]];
n24l4 = Get[StringJoin["d", d, "ml4n24l4res"]];
ndn24l1 = Get[StringJoin["d", d, "ml4ndn24l1res"]];
ndn24l2 = Get[StringJoin["d", d, "ml4ndn24l2res"]];
ndn24l3 = Get[StringJoin["d", d, "ml4ndn24l3res"]];
ndn24l4 = Get[StringJoin["d", d, "ml4ndn24l4res"]];

dln24m2 = Prepend[1 - glmm2[[3 ;; 5, sn + 1]], Null ];
dln24ci = 1 - {Count[n24l1[[All, 2]], False], Count[n24l2[[All, 2]], False],
      Count[n24l3[[All, 2]], False], Count[n24l4[[All, 2]], False]} / 1000;
dln24t = 1 - {Count[n24l1[[All, 4]], False], Count[n24l2[[All, 4]], False],
      Count[n24l3[[All, 4]], False], Count[n24l4[[All, 4]], False]} / 1000;
dln24anova = 1 - {Count[n24l1[[All, 6]], False], Count[n24l2[[All, 6]], False],
      Count[n24l3[[All, 6]], False], Count[n24l4[[All, 6]], False]} / 1000;
dln24anovax = 1 - {Count[n24l1[[All, 8]], False], Count[n24l2[[All, 8]], False],
      Count[n24l3[[All, 8]], False], Count[n24l4[[All, 8]], False]} / 1000;

nddln24m2 = Prepend[1 - glmm2n[[3 ;; 5, sn + 1]], Null ];
nddln24ci = 1 - {Count[ndn24l1[[All, 2]], False], Count[ndn24l2[[All, 2]], False],
      Count[ndn24l3[[All, 2]], False], Count[ndn24l4[[All, 2]], False]} / 1000;
nddln24t = 1 - {Count[ndn24l1[[All, 4]], False], Count[ndn24l2[[All, 4]], False],
      Count[ndn24l3[[All, 4]], False], Count[ndn24l4[[All, 4]], False]} / 1000;
nddln24anova = 1 - {Count[ndn24l1[[All, 6]], False], Count[ndn24l2[[All, 6]], False],
      Count[ndn24l3[[All, 6]], False], Count[ndn24l4[[All, 6]], False]} / 1000;
nddln24anovax = 1 - {Count[ndn24l1[[All, 8]], False], Count[ndn24l2[[All, 8]], False],
      Count[ndn24l3[[All, 8]], False], Count[ndn24l4[[All, 8]], False]} / 1000;

p24 =
```

```
  ListLinePlot[{dor[{d1n24m2, ndd1n24m2}] //. 1 → Null, dor[{d1n24ci, ndd1n24ci}],
    dor[{d1n24t, ndd1n24t}], dor[{d1n24anova, ndd1n24anova}],
    dor[{d1n24anovax, ndd1n24anovax}]}, ScalingFunctions → "Log",
   Frame -> True, AspectRatio → 1, PlotRange → {{0.5, 4.5}, {0.1, 1000}},
   FrameTicks → {{{0.1, 0.1, 1, 10, 100, 1000}, None}, {{1, 2, 3, 4}, None}},
   FrameTicksStyle → Directive[Black, 20]];

n48l1 = Get[StringJoin["d", d, "ml4n48l1res"]];
n48l2 = Get[StringJoin["d", d, "ml4n48l2res"]];
n48l3 = Get[StringJoin["d", d, "ml4n48l3res"]];
n48l4 = Get[StringJoin["d", d, "ml4n48l4res"]];
ndn48l1 = Get[StringJoin["d", d, "ml4ndn48l1res"]];
ndn48l2 = Get[StringJoin["d", d, "ml4ndn48l2res"]];
ndn48l3 = Get[StringJoin["d", d, "ml4ndn48l3res"]];
ndn48l4 = Get[StringJoin["d", d, "ml4ndn48l4res"]];

d1n48m2 = Prepend[1 - glmm2[[6 ;; 8, sn + 1]], Null ];
d1n48ci = 1 - {Count[n48l1[[All, 2]], False], Count[n48l2[[All, 2]], False],
     Count[n48l3[[All, 2]], False], Count[n48l4[[All, 2]], False]} / 1000;
d1n48t = 1 - {Count[n48l1[[All, 4]], False], Count[n48l2[[All, 4]], False],
     Count[n48l3[[All, 4]], False], Count[n48l4[[All, 4]], False]} / 1000;
d1n48anova = 1 - {Count[n48l1[[All, 6]], False], Count[n48l2[[All, 6]], False],
     Count[n48l3[[All, 6]], False], Count[n48l4[[All, 6]], False]} / 1000;
d1n48anovax = 1 - {Count[n48l1[[All, 8]], False], Count[n48l2[[All, 8]], False],
     Count[n48l3[[All, 8]], False], Count[n48l4[[All, 8]], False]} / 1000;

ndd1n48m2 = Prepend[1 - glmm2n[[6 ;; 8, sn + 1]], Null ];
ndd1n48ci = 1 - {Count[ndn48l1[[All, 2]], False], Count[ndn48l2[[All, 2]], False],
     Count[ndn48l3[[All, 2]], False], Count[ndn48l4[[All, 2]], False]} / 1000;
ndd1n48t = 1 - {Count[ndn48l1[[All, 4]], False], Count[ndn48l2[[All, 4]], False],
     Count[ndn48l3[[All, 4]], False], Count[ndn48l4[[All, 4]], False]} / 1000;
ndd1n48anova = 1 - {Count[ndn48l1[[All, 6]], False], Count[ndn48l2[[All, 6]], False],
     Count[ndn48l3[[All, 6]], False], Count[ndn48l4[[All, 6]], False]} / 1000;
ndd1n48anovax = 1 - {Count[ndn48l1[[All, 8]], False], Count[ndn48l2[[All, 8]], False],
     Count[ndn48l3[[All, 8]], False], Count[ndn48l4[[All, 8]], False]} / 1000;

p48 =
  ListLinePlot[{dor[{d1n48m2, ndd1n48m2}] //. 1 → Null, dor[{d1n48ci, ndd1n48ci}],
    dor[{d1n48t, ndd1n48t}], dor[{d1n48anova, ndd1n48anova}],
    dor[{d1n48anovax, ndd1n48anovax}]}, ScalingFunctions → "Log",
   Frame -> True, AspectRatio → 1, PlotRange → {{0.5, 4.5}, {0.1, 1000}},
   FrameTicks → {{{0.01, 0.1, 1, 10, 100, 1000}, None}, {{1, 2, 3, 4}, None}},
   FrameTicksStyle → Directive[Black, 20]];
cgodd1 = GraphicsGrid[{{p12, p24, p48}}, ImageSize → 800]
```

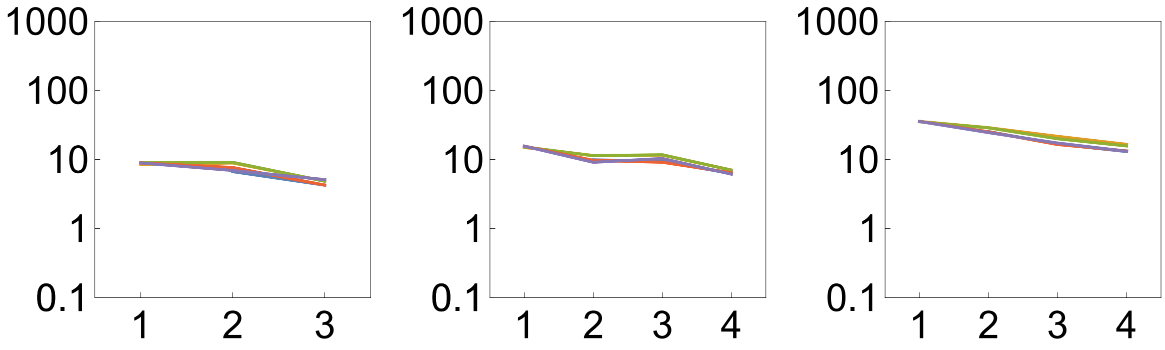

Lines: blue: GLMM: treatment+(1 | lab), violet: ANOVA x+y+xy, red: ANOVA x+y, green: pooled t-test, yellow: CI95%

4. Power

```
trueesh = 0.4781;

trueeslist = {0.1978, 0.4602, 0.3896, 0.4749, 0.3446,
   -0.5174, 0.3593, 0.2454, 0.3575, 0.5117, -0.3035, 0.1913};

originalpower =
 Table[power[{Mean[data[[All, 4]] / data[[All, 4]]], Mean[data[[All, 5]] / data[[All, 4]]],
    Mean[data[[All, 6]] / data[[All, 4]]], Mean[data[[All, 7]] / data[[All, 4]]],
    data[[i, 2]], data[[i, 3]]}], {i, nos}]

powers12 =
 Table[power[{data[[i, 4]], data[[i, 5]], data[[i, 6]], data[[i, 7]], 12, 12}], {i, nos}]

powers24 =
 Table[power[{data[[i, 4]], data[[i, 5]], data[[i, 6]], data[[i, 7]], 24, 24}], {i, nos}]

powers48 =
 Table[power[{data[[i, 4]], data[[i, 5]], data[[i, 6]], data[[i, 7]], 48, 48}], {i, nos}]
```
